# Sampling weighting strategies in causal mediation analysis

**DOI:** 10.1186/s12874-024-02262-x

**Published:** 2024-06-15

**Authors:** Donna L. Coffman, Haoyu Zhou, Katherine E. Castellano, Megan S. Schuler, Daniel F. McCaffrey

**Affiliations:** 1 Department of Psychology, University of South Carolina, 1512 Pendleton St., Columbia, 29208 USA; 2 Department of Epidemiology and Biostatistics, Temple University, Philadelphia, USA; 3 RAND, Arlington, USA; 4 Educational Testing Service, Princeton, USA

**Keywords:** Mediation analysis, Sampling weights, Propensity scores

## Abstract

**Background:**

Causal mediation analysis plays a crucial role in examining causal effects and causal mechanisms. Yet, limited work has taken into consideration the use of sampling weights in causal mediation analysis. In this study, we compared different strategies of incorporating sampling weights into causal mediation analysis.

**Methods:**

We conducted a simulation study to assess 4 different sampling weighting strategies-1) not using sampling weights, 2) incorporating sampling weights into mediation “cross-world” weights, 3) using sampling weights when estimating the outcome model, and 4) using sampling weights in both stages. We generated 8 simulated population scenarios comprising an exposure (*A*), an outcome (*Y*), a mediator (*M*), and six covariates (*C*), all of which were binary. The data were generated so that the true model of A given C and the true model of A given M and C were both logit models. We crossed these 8 population scenarios with 4 different sampling methods to obtain 32 total simulation conditions. For each simulation condition, we assessed the performance of 4 sampling weighting strategies when calculating sample-based estimates of the total, direct, and indirect effects. We also applied the four sampling weighting strategies to a case study using data from the National Survey on Drug Use and Health (NSDUH).

**Results:**

Using sampling weights in both stages (mediation weight estimation and outcome models) had the lowest bias under most simulation conditions examined. Using sampling weights in only one stage led to greater bias for multiple simulation conditions.

**Discussion:**

Using sampling weights in both stages is an effective approach to reduce bias in causal mediation analyses under a variety of conditions regarding the structure of the population data and sampling methods.

**Supplementary Information:**

The online version contains supplementary material available at 10.1186/s12874-024-02262-x.

## Background

An important scientific goal in many fields of research is determining to what extent the effect of an exposure on an outcome is mediated by an intermediate variable on the causal pathway between the exposure and outcome. In mediation analysis, the effect of an exposure on outcome is decomposed into an indirect effect, the effect that is mediated through the intermediate variable, and a direct effect, the difference between the overall or total effect and the indirect effect. Traditionally, mediated effects have been evaluated using linear model specifications for the observed data [1]. The definitions of the direct and indirect effects themselves rely on this linear specification. Robins and Greenland [2] and Pearl [3] developed fully-nonparametric causal models for defining, identifying, and estimating direct and indirect effects that do not rely on a linear model specification. This approach to mediation analysis is commonly referred to as “causal mediation modeling” because it uses potential outcomes/counterfactuals used in causal modeling for treatment effects to give non-parametric definitions of the effects involved in mediation analysis.

An underlying assumption of causal mediation analysis is that the data are a simple random sample from the population of interest. However, often this assumption does not hold. In survey samples, individuals are sampled randomly but with unequal probabilities. Alternatively, analytic samples may not reflect a random sample due to missingness or attrition. A common approach to adjust for nonrandom sampling is to weight the sample such that the sample matches the population on observed covariates. Prior methodological work has considered causal modeling with nonrandom sampling for the effects of dichotomous treatments [4] or continuous treatments [5], but there has been little study of how to conduct causal mediation analysis on nonrandom samples.

We address this issue by assessing how best to use weights to account for nonrandom sampling (i.e., arising from survey sampling, nonresponse, or attrition) when conducting causal mediation analysis. We next provide a brief review of the literature on the use of sampling, attrition, or nonresponse weights for causal modeling, in general, followed by an introduction to causal mediation analysis with random samples. We then detail our proposed approach to causal mediation analysis with nonrandom sampling and present the results of our simulation study. We conclude with an applied example of our proposed approach in which we examine potential mediating pathways underlying substance use disparities among sexual minority (e.g., gay, lesbian, or bisexual) women, using survey data from the National Survey of Drug Use and Health (NSDUH).

## Sampling weights and causal modeling

Multiple authors have considered the use of sampling weights for causal modeling with binary treatments (e.g., Zanutto [6]; DuGoff et al. [7]; Ridgeway et al. [4]; Austin et al. [8]; Lenis et al. [9]; Dong et al. [10]), yielding varying suggestions for how best to use sampling weights when estimating treatment effects. Zanutto [6] focused on propensity score (PS) stratification, recommending in that context that sampling weights only be used in the outcome analyses estimating stratum-specific treatment effects. Zanutto argued that the PS model did not need sampling weights, as this model was used to create strata of individuals with similar propensities rather than population-level inferences. DuGoff et al. [7] broadened their focus to PS matching, weighting, and stratification and recommended that, in addition to applying sampling weights in the outcome models, sampling weights should be used as a covariate (rather than as weights) in the PS model. Ridgeway et al. [4] focused on PS weighting and, in contrast to the first two papers, used theoretical derivation to show that consistent estimation of the treatment effects for the population can be obtained by (1) using sampling weights (as weights, not a covariate) in the estimation of the PS model and (2) weighting the outcome model by the product of the sampling weights and inverse probability weights (IPW). Via simulations they also showed that this method resulted in better covariate balance and treatment effect estimates with the lowest root mean squared error (RMSE) across several data generating scenarios (including the ones considered in DuGoff et al. [7]). Austin et al. [8] and Lenis et al. [9] both explored the implications of different approaches for handling sampling weights for PS matching and generally showed that no method of estimation was clearly preferable to the others. McCaffrey et al. [5] extended this research to continuous exposures. Like Ridgeway et al. [4], they showed that using sampling weights in the estimation of the generalized propensity score (GPS) model and weighting by the product of the sampling weights and inverse GPS weights when estimating the treatment effects is sufficient for consistent estimation of the treatment effects for the population. They also showed that under various scenarios using sampling weights in both estimation stages is not necessary and that this held for multiple simulation study conditions. However, this issue has not been examined in the mediation context.

## Background on causal mediation

**Fig. 1:**
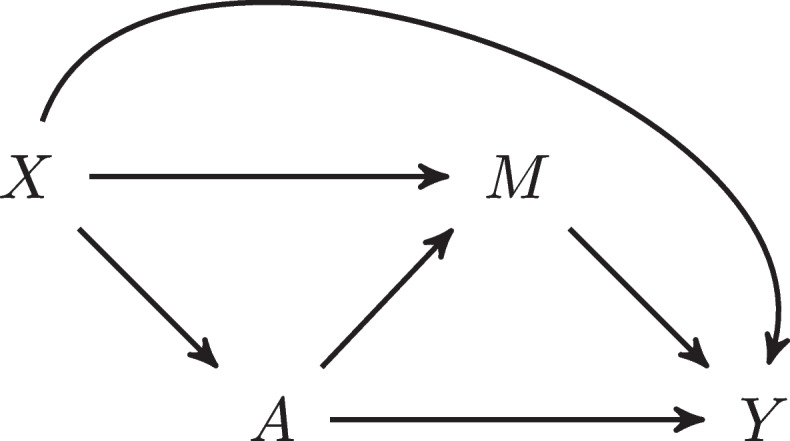
Graphical depiction of a simple mediation model

A simple mediation model is illustrated in Fig. 1 where *Y* represents the outcome, *A* represents the exposure, *X* represents pre-exposure covariates, and *M* denotes the mediator. Note that we use the term “exposure” broadly to refer to a non-randomized or randomized condition, treatment, or intervention.

The total effect of *A* on *Y* includes two possible causal paths from *A* to *Y*: the path $A \rightarrow M \rightarrow Y$ is the indirect effect of *A* on *Y* through *M* and the path $A \rightarrow Y$ is the direct effect of *A* on *Y* that does not go through *M*. Mediation is inherently about causal effects, which are defined as the difference between two potential outcomes for an individual. We begin by introducing the potential outcomes needed to define the natural direct and indirect effects.

Consider the case in which *A* is a binary indicator of the exposure, indicating the exposed condition ($A=1$) or the comparison condition ($A=0$). There are two potential outcomes for each study participant corresponding to each exposure level *a*: the outcome had they received the exposure, denoted $Y_1$, and the outcome had they received the comparison condition, denoted $Y_0$. These two potential outcomes, $Y_1$ and $Y_0$, exist for all individuals in the population regardless of whether the individual received the exposure or comparison condition. However, we can only observe one of these outcomes for each participant depending on which exposure condition the individual actually receives.

The mediator is an “intermediate” outcome of the exposure and itself has potential values. For each exposure level *a* there is a corresponding potential mediator value, denoted $M_a$. Also, there is a corresponding potential outcome that reflects the outcome value that would arise under the specific exposure level *a* and the specific potential mediator value $M_a$ – this potential outcome is denoted $Y_{(a, M_a)}$. Causal definitions of direct and indirect effects require extending the potential outcomes framework such that there is a potential outcome for each treatment and mediator pair. For the case of a binary exposure *A*, there are four potential outcomes for an individual, formed by crossing both potential exposure values with both potential mediator values: $Y_{(1, M_1)}$, $Y_{(0, M_0)}$, $Y_{(1, M_0)}$, and $Y_{(0, M_1)}$. Only $Y_{(1, M_1)}$ or $Y_{(0, M_0)}$ are observable, as these are the only potential outcomes for which the exposure status is concordant with the exposure status for the mediator potential value. The other two potential outcomes (often referred to as *cross-world counterfactuals* or *cross-world potential outcomes*) are hypothetical quantities because the mediator value is manipulated to take on the value it would have under the other exposure condition; these cross-world counterfactuals are necessary to define the causal estimands of interest. In practice, we can only observe one outcome for a given individual *i* – either $Y_{(1, M_1)}$ or $Y_{(0, M_0)}$ – corresponding to the exposure level *a* that the individual actually received.

### Estimands: natural direct and indirect effects

Causal effects are defined as contrasts between different potential outcomes. Specifically, we consider the natural direct and natural indirect effects. Alternative mediation estimands exist (see [11] for details), but the natural direct and indirect effects tend to be used in most applications.

As with causal modeling for (non-mediated) treatment effects, the total effect of a dichotomous exposure for an individual equals the difference between the potential outcome when exposed and the potential outcome when not exposed or $Y_{(1, M_1)} - Y_{(0, M_0)}$. The estimand of interest for the total effect is the expected value of the individual effects across the entire population:

1 $$\begin{aligned} TE = E\left[ Y_{(1, M_1)} - Y_{(0, M_{0})} \right] = E\left[ Y_{1} - Y_{0} \right] . \end{aligned}$$

The natural direct effect (NDE) and natural indirect effect (NIE) are defined as follows:

2 $$\begin{aligned} NDE_{0}{} & {} = E\left[ Y_{(1, M_{0})} - Y_{(0, M_{0})} \right] \end{aligned}$$

3 $$\begin{aligned} NIE_1{} & {} = E\left[ Y_{(1, M_1)} - Y_{(1, M_{0})} \right] . \end{aligned}$$

We can similarly define an alternative TE decomposition comprised of $NDE_1$ and $NIE_0$:

4 $$\begin{aligned} NDE_{1}{} & {} = E\left[ Y_{(1, M_{1})} - Y_{(0, M_{1})} \right] \end{aligned}$$

5 $$\begin{aligned} NIE_0{} & {} = E\left[ Y_{(0, M_1)} - Y_{(0, M_{0})} \right] . \end{aligned}$$

There are two decompositions for *TE*: $TE = NDE_{0} + NIE_1$ and $TE = NDE_{1} + NIE_0$. The subscripts for *NDE* (i.e., $NDE_{0}$ or $NDE_{1}$) denote the condition to which the mediator is held constant, whereas the subscripts for *NIE* denote the condition to which the exposure is held constant. We note that each decomposition includes an *NIE* and an *NDE* corresponding to opposite subscripts.

### Identification assumptions

Identification of the natural indirect and direct effects relies on the assumptions of positivity, consistency, and sequential ignorability. First, the positivity assumption requires that all individuals have some positive probability of receiving each level of the exposure and each level of the mediator. Second, the consistency assumption states that the outcome observed for an individual is identical to (i.e., consistent with) the potential outcome that corresponds to their observed exposure value; similarly, their observed mediator value is the potential mediator value that corresponds to their observed exposure value. Finally, sequential ignorability refers to a set of assumptions regarding confounding. The nonparametric assumptions typically made for identification of NDE and NIE conditional on pre-exposure variables *X* are the following:

1. No unobserved confounding of the effect of *A* on *M*
2. No unobserved confounding of the effect of *A* on *Y*
3. No unobserved confounding of the effect of *M* on *Y*
4. No confounder (observed or unobserved) of the effect of *M* on *Y* that is affected by *A*

If individuals are randomly assigned to levels of the exposure, then assumptions 1 and 2 should hold. However, assumptions 3 and 4 may not hold even when there is random assignment to the exposure. See [12] for further discussion of these identifying assumptions. Implicit in these identifying assumptions is the assumption that data are a simple random sample of the population.

### Estimation

The basic idea is to obtain estimates of $E\left[ Y_{(1,M_1)}\right]$, $E\left[ Y_{(0,M_0)}\right]$, $E\left[ Y_{(1,M_0)}\right]$, and $E\left[ Y_{(0,M_1)}\right]$ which are then plugged into Eqs. 2 and 3 or 4 and 5 to obtain estimates of the natural indirect and direct effects. Although there are numerous estimation strategies, we will focus on weighting because it involves estimation of weights and the question arises as to whether the sampling weights should be included in the estimation of these weights, just as the question arose for weighted estimators of dichotomous or continuous treatments. Hong [13] first defined the following weights $w_{aa'}$ to estimate each potential outcome, $E\left[ Y_{(a,M_{a'})}\right]$ for $a \in (0,1)$ and $a' \in (0,1)$:

6 $$\begin{aligned} w_{aa'} = \frac{f(M=m | A=a', X=x)}{f(M=m | A=a, X=x)p(A=a | X=x)} \end{aligned}$$

where *f*(.) is a density function. Note that $w_{aa'}$ is a function of *X* as well as *a* and $a'$, but we omit *X* from the $w_{aa'}$ notation for simplicity. The estimate of $E\left[ Y_{(a,M_{a'})}\right]$ is the weighted mean,

$$\begin{aligned} E\left[ \hat{Y}_{(a,M_{a'})}\right] = \sum _{i: A_i = a} Y^{obs}_i w_{aa',i} / \sum _{i: A_i = a} w_{aa',i}. \end{aligned}$$

Under the previously stated assumptions of consistency, positivity, and sequential ignorability (i.e., *X* strictly pre-exposure, or not affected by *A*), Huber [14] used the following manipulation (i.e., Bayes Rule)

$$\begin{aligned} f(M=m | A =a, X=x) = \frac{p(A=a | M=m, X=x) f(M=m | X=x)}{p(A=a | X=x)} \end{aligned}$$

to obtain an easier set of weights to estimate:

7 $$\begin{aligned} w_{aa'}{} & {} = \frac{p(M=m|A=a', X=x)}{p(M =m | A=a, X=x) p(A=a | X=x)}\end{aligned}$$

8 $$\begin{aligned}{} & {} = \overset{\text {Odds Weight}}{\overbrace{\frac{p(A=a'|M=m, X=x)}{p(A=a|M=m, X=x)}}} \overset{\text {IPW}}{\overbrace{\frac{1}{p(A = a'|X = x)}}} \end{aligned}$$

These weights have been referred to as “cross-world” weights as they are used to estimate the average cross-world potential outcomes (i.e., $E\left[ Y_{(1, M_0)}\right]$ or $E\left[ Y_{(0, M_1)}\right]$) [15]. In the denominator of Equation 8, note that $p(A=a|X=x)$ appears on the left hand side whereas $p(A=a'|X=x)$ appears on the right hand side; the change is the result of applying Bayes rule for the numerator and denominator of Eq. 6. Following [15], we will refer to the first term comprising the product on the right hand side of Eq. 8 as an *odds weight* and the second term as an *inverse probability weight* (IPW). These terms are so named because the IPW is of the standard IPW form and the odds weight term is the usual form for estimating the average treatment effect among the treated/exposed (ATT), with the addition of conditioning on the mediator. In practice, the odds weight and IPW are calculated separately and then multiplied together to obtain the final cross-world weights. Since $E[Y_{(1,M_1)}]$ is the expected value of the potential outcome under treatment, $w_{11}=1/p(A=1 \mid X = x)$. Similarly, $w_{00}=1/p(A=0 \mid X = x)$.

The development of these cross-world weights assumes the data are a simple random sample from the population of interest. The following section details our proposed approach for generalizing cross-world weights to incorporate sampling weights.

## Accounting for sampling weights in cross-world potential outcomes

Our proposed approach is an extension of prior theoretical work that showed that the use of composite weights – generated by multiplying sampling weights by IPW or generalized propensity score weights – yielded consistent estimation of population-level treatment effects [4, 5]. In a similar manner, we propose to create composite weights that are generated by multiplying sampling weights by cross-world weights for mediation analyses.

Below, we provide a theoretical derivation showing that the proposed composite weights will yield consistent estimates of population-level natural direct and indirect effects in a sample. We assume that all the standard assumptions for causal mediation hold and that the mediator *M* and exposure *A* are sequentially ignorable given *X*. Sampling selection is not random, but it is random conditional on the sample design variables, denoted *U*. Let *Z* denote a sample selection indicator in which $Z=1$ for sampled individuals and 0 otherwise; thus, $Z=1$ for all individuals in the sample. The outcome is denoted *Y* and the observed data are $(Y^{obs}_i,M^{obs}_i,X_i,U_i,A_i,Z_i)$, $i=1, \ldots , n$. We assume $Y^{obs}=Y(A,M(A))$ and $M^{obs}=M(A)$. Let *S* denote an indicator for whether an individual was selected into a sample, with $S(U)=P(Z=1)/P(Z = 1 \mid U)$ and $S_i = S(U_i)$ and $W(X) = \frac{P(A = 0 \mid M^{obs}, X)}{P(A= 1 \mid M^{obs}, X)P(A=0 \mid X)}$ and $W_i = W(X_i)$. It is possible that we observe $S_i$ and not $U_i$.

In addition to sequential ignorability, we assume $Y^{obs}, M^{obs},X, A {\perp \!\!\!\perp } Z \mid U$. We now show that $E[Y^{obs},W,S \mid Z=1, A=1 ] = E[Y(1,M(0))]$

9 $$\begin{aligned}{} & {} = \int \dots \int W(X)S(U)y \times \nonumber \\{} & {} \qquad \qquad \qquad \quad f_{Y^{obs},M^{obs},X,U \mid A=1,Z=1}(y,m,X,U \mid A=1, Z=1) dy \, dm \, dX \, dU \nonumber \\{} & {} = \int \dots \int W(X)S(U)y a\times \nonumber \\{} & {} \qquad \qquad \qquad \quad f_{Y^{obs},M^{obs},X,U,A \mid Z=1}(y,m,X,U,a \mid Z=1) dy \, dm \, dX \, dU \, da \nonumber \\{} & {} = \int \dots \int W(X)S(U)y af_{Y^{obs},M^{obs},X,A \mid U, Z=1}(y,m,X,a \mid U,Z=1)\times \nonumber \\{} & {} \qquad \qquad \qquad \quad f_{U \mid Z}(U \mid Z=1)dy \, dm \, dX \, dU \, da \nonumber \\{} & {} = \int \dots \int W(X)S(U)y af_{Y^{obs},M^{obs},X,A \mid U}(y,m,X,a \mid U)\times \nonumber \\{} & {} \qquad \qquad \qquad \quad f_{U \mid Z}(U \mid Z=1)dy \, dm \, dX \, dU \, da \nonumber \\{} & {} = \int \dots \int W(X)S(U)y af_{Y^{obs},M^{obs},X,A \mid U}(y,m,X,a \mid U)\times \nonumber \\{} & {} \qquad \qquad \qquad \quad \frac{f_{Z \mid U}(Z=1 \mid U)f_U(U)}{P(Z=1)}dy \, dm \, dX \, dU \, da \nonumber \\{} & {} = \int \dots \int W(X)y af_{Y^{obs},M^{obs},X,A \mid U}(y,m,X,a \mid U)\times \nonumber \\{} & {} \qquad \qquad \qquad \quad f_U(U)dy \, dm \, dX \, dU \, da \nonumber \\{} & {} = \int \dots \int W(X)y af_{Y^{obs},M^{obs},X,A}(y,m,X,a)dy \, dm \, dX \, da \nonumber \\{} & {} = \int \dots \int W(X)y f_{Y^{obs},M^{obs},X \mid A=1 }(y,m,X \mid A=1)dy \, dm \, dX \end{aligned}$$

The results follow using the standard derivations for cross-world weighting. Note that since $U \ne X$, the *W*(*X*) are for the population and are not conditional on *Z*. However, we can only estimate $P(A = 1 \mid M^{obs}, X)$ and $P(A = 0 \mid X)$ using the observed sample. We can estimate the conditional probabilities for the population using the observed sample provided we weight by the sampling weights when estimating the models [16]. Hence, we need to use sampling weights both when estimating $P(A = 1 \mid M^{obs}, X)$ and $P(A = 0 \mid X)$ and as part of the composite with the cross-world weights. These results apply regardless of how individuals are selected for the sample, whether it is by a probability sample design or by providing complete data or remaining enrolled in the study. As long as the selection mechanism is ignorable conditional on *U* and *S*(*U*) equals the inverse of the probability of selection, the result will hold.

To summarize, there has been a question in previous research as to whether sampling weights should be included in estimating the propensity model in the context of both binary and continuous treatments. In the causal mediation literature, this question extends to estimation of the weights for weighting estimators of causal mediation effects. That is, do the sampling weights need to be included in estimating the weights (i.e., a propensity model), or do they only need to be included in the weighted outcome analysis. Although there are other estimators of causal mediation effects, this question does not directly apply to them because they do not involve estimation of weights (i.e., a propensity model) and thus, we do not include those estimators in the simulation study.

## Simulation study

We conducted a simulation study to evaluate the performance of composite weights that equal the product of the sampling and cross-world weights, varying whether sampling weights are used when estimating the conditional probabilities of exposure. We also compare composite weighting approaches to a naïve approach of not using sampling weights in either stage. Our simulations consider a range of different populations scenarios defined by increasingly complex associations among the variables of interest as well as several different sampling methods used to select the sample population from the total population.

### Data generation

We generated eight population scenarios of 90,000 individuals each. These scenarios were chosen to represent a range of complexity in the relationships among the variables of interest. Each scenario comprised the following variables (all of which were dichotomous): the exposure *A*, a mediator *M*, the outcome *Y*, and six covariates: $U_1$, $U_2$, $U_3$, $X_1$, $X_2$, $X_3$. The set of six covariates were divided into two groups: variables related to sampling selection (i.e., the *U*s) and those not related to sampling selection (i.e., the *X*s). All six covariates, collectively denoted simply as *C*, were related to *A*, *M*, and *Y*.

To add complexity to the data distribution, we divided the population into three strata. Each stratum made up 1/3 of the population, and we generated the covariates, *C*, according to the following distributions:

- Stratum 1: $U_1 \sim$ Bernoulli(0.3), $U_2 \sim$ Bernoulli(-0.4), $U_3 \sim$ Bernoulli(-0.2), $X_1 \sim$ Bernoulli(0.25), $X_2 \sim$ Bernoulli(0.2), $X_3 \sim$ Bernoulli(-0.3).
- Stratum 2: $U_1 \sim$ Bernoulli(0.5), $U_2 \sim$ Bernoulli(0), $U_3 \sim$ Bernoulli(0.1), $X_1 \sim$ Bernoulli(0.6), $X_2 \sim$ Bernoulli(0.4), $X_3 \sim$ Bernoulli(-0.1).
- Stratum 3: $U_1 \sim$ Bernoulli(0.7), $U_2 \sim$ Bernoulli(0.4), $U_3 \sim$ Bernoulli(0.2), $X_1 \sim$ Bernoulli(0.75), $X_2 \sim$ Bernoulli(0.8), $X_3 \sim$ Bernoulli(0.3).

The strata only affect the generation of the covariates, *U* and *X*, and as described below, they do not affect the sampling procedures or the relationships between the covariates and any of the other variables–exposure, mediator, and outcome. Thus, they are not included in any analyses.

The eight population scenarios had the following associations between *A*, *M*, *C*, and *Y*. Scenario 1 was the simplest one where the true models that link *A*, *C*, and *M* had only main effects of the variables and no interactions. In contrast, Scenarios 2 to 4 added an interaction term to the model for *Y*; Scenario 5 included an interaction term in the model for *M*; Scenarios 6 through 8 each included an interaction term in both the models for *M* and *Y*.

1. Scenario 1: *A* $$\sim$$ *C*, *M* $$\sim$$ *A* + *C*, *Y* $$\sim$$ *A* + *M* + *C*;
2. Scenario 2: *A* $$\sim$$ *C*, *M* $$\sim$$ *A* + *C*, *Y* $$\sim$$ *A* + *M* + $$A \times M$$ + *C*;
3. Scenario 3: *A* $$\sim$$ *C*, *M* $$\sim$$ *A* + *C*, *Y* $$\sim$$ *A* + *M* + *C* + $$A \times X_2$$;
4. Scenario 4: *A* $$\sim$$ *C*, *M* $$\sim$$ *A* + *C*, *Y* $$\sim$$ *A* + *M* + *C* + $$M \times X_2$$;
5. Scenario 5: *A* $$\sim$$ *C*, *M* $$\sim$$ *A* + *C* + $$A \times X_2$$, *Y* $$\sim$$ *A* + *M* + *C*;
6. Scenario 6: *A* $$\sim$$ *C*, *M* $$\sim$$ *A* + *C* + $$A \times X_2$$, *Y* $$\sim$$ *A* + *M* + $$A \times M$$ + *C*;
7. Scenario 7: *A* $$\sim$$ *C*, *M* $$\sim$$ *A* + *C* + $$A \times X_2$$, *Y* $$\sim$$ *A* + *M* + *C* + $$A \times X_2$$;
8. Scenario 8: *A* $$\sim$$ *C*, *M* $$\sim$$ *A* + *C* + $$A \times X_2$$, *Y* $$\sim$$ *A* + *M* + *C* + $$M \times X_2$$.

The data were defined in a way such that the true model of treatment assignment *A* given the covariates *C*, and the true model of *A* given *M* and *C* were both logit models. *P*(*M*|*A*, *C*) was determined by these two models. Specifically, we created three vectors *a*, *b*, and *d*, which were defined by the formulas below:

$$\begin{aligned} a{} & {} = 0.68 + \alpha 'C\\ b{} & {} = 0.8 + \beta 'C\\ d{} & {} = -1.52 + \delta X_2 \end{aligned}$$

Specifically, $\alpha$ and $\beta$ represented vectors of parameters of length seven (one constant and six covariate coefficients). The equation for *d* was used to determine if an interaction existed between *M* and $X_2$ in the true model of *A* given *M* and *C*; $\delta$ = -0.5 if the interaction between *M* and $X_2$ existed in the true model of *A* given *M* and *C*, and 0 otherwise. We then generated the exposure *A* and the mediator *M* using the probabilities defined as:

10 $$\begin{aligned} P(A = 1){} & {} = 1/(1+exp(-a))\end{aligned}$$

11 $$\begin{aligned} P(A = 1|M = 0){} & {} = 1/(1+exp(-b))\end{aligned}$$

12 $$\begin{aligned} P(A = 1|M = 1){} & {} = 1/(1+exp(-b-d)) \end{aligned}$$

With the unconditional and conditional probabilities of $A = 1$ defined above, we can derive the conditional probabilities, $P(M \vert A = 1)$, as follows:

13 $$\begin{aligned} P(M = 1 | A = 1){} & {} = (exp(-b)-exp(-a)) / (exp(-b)-exp(-b-d))\end{aligned}$$

14 $$\begin{aligned} P(M = 1 | A = 0){} & {} = (exp(-b)-exp(-a)) / (exp(d-a)-exp(-a)) \end{aligned}$$

### True effects

We calculated the true total effects (*TE*), natural direct effects (*NDE*), and natural indirect effects (*NIE*) using pseudo-populations that represented four potential outcomes – $Y_{(1, M_1)}$, $Y_{(0, M_0)}$, $Y_{(1, M_0)}$, $Y_{(0, M_1)}$ – according to the consistency assumption. The true *TE*, *NDE*, and *NIE* were calculated by subtraction between the corresponding pseudo-population means.

### Sampling and estimation

For each simulation condition, we drew a sample of 9,000 individuals from the total population of 90,000 individuals; this process was replicated 1,000 times per simulation condition. To reflect a variety of sampling strategies that may occur in practice, we examined **4 sampling methods**:

1. Sampling depends on covariates only: *S* $\sim$ *U*;
2. Sampling depends on covariates and the mediator: *S* $\sim$ *U* + *M*;
3. Sampling depends on covariates and the treatment: *S* $\sim$ *U* + *A*;
4. Simple random sampling: $S \sim 1$

*S* is a binary variable determining whether an individual was selected into a sample in an iteration; *U* denotes the vector of covariates ($U_1$, $U_2$, $U_3$). We conducted a fully crossed simulation study with 32 conditions (eight population scenarios $\times$ four sampling methods).

We tested **4 sampling weighting strategies**:

1. Naïve: No sampling weights used in any part of the analysis
2. Using sampling weights only in outcome models (second stage)
3. Using sampling weights only in estimation of mediation cross-world weights (first stage)
4. Using sampling weights in both stages

All four weighting strategies were applied in the mediation analysis of the 32 conditions. For each of the 1,000 replicate samples for each of the 32 simulation conditions, we performed the following analytic steps.

1. All analyses used propensity score weighting to account for the nonrandom assignment of treatment. We estimated the propensity score by fitting a logistic regression model to predict *A* from covariate vector *C*. Estimated propensity scores were used to generate IPW weights which were used both in estimating cross-world mediation weights and when estimating outcome models. Under sampling weighting strategies 3 and 4, sampling weights were incorporated into estimation of the propensity scores and mediation weights.
2. All analyses used weighting to estimate TE, NIE, and NDE. Mediation weights were estimated using the wgtmed function from the R package twangMediation [17].
3. Outcome logistic regression models were used to estimate weighted group means (i.e., $E[Y_{(1, M_1)}]$, $E[Y_{(0, M_0)}]$, $E[Y_{(0, M_1)}]$, and $E[Y_{(1, M_0)}]$) which were used to calculate treatment effects-*TE*, $NDE_1$, $NIE_0$, $NDE_0$, and $NIE_1$. Under sampling weighting strategies 2 and 4, outcome models used sampling weights.

For each condition and sampling weighting strategy, we calculated the following summary statistics over the 1,000 replicate samples for each of the five effect estimates (*TE*, $NIE_1$, $NDE_0$, $NIE_0$, and $NDE_1$) and the four potential outcomes ($Y_{(1, M_1)}$, $Y_{(1, M_0)}$, $Y_{(0, M_1)}$, $Y_{(0, M_0)}$): mean, bias (average difference between estimates and true effects calculated based on potential outcomes in the population), relative bias (bias divided by the true effect), mean squared error between the estimates and true effects for simulating the data (MSE), mean of the estimated standard error (SE), and standard deviation (SD) of the estimates across the 1000 replications.

### Results

We evaluated the recovery of the population effects for each of the four sampling weighting strategies for each of the 32 simulation conditions examined. For simplicity, we only present the results under the simplest scenario (Scenario 1) and one of the more complex scenarios (Scenario 8). The results for the other six scenarios are generally similar and are available in Appendix A.

**Fig. 2:**
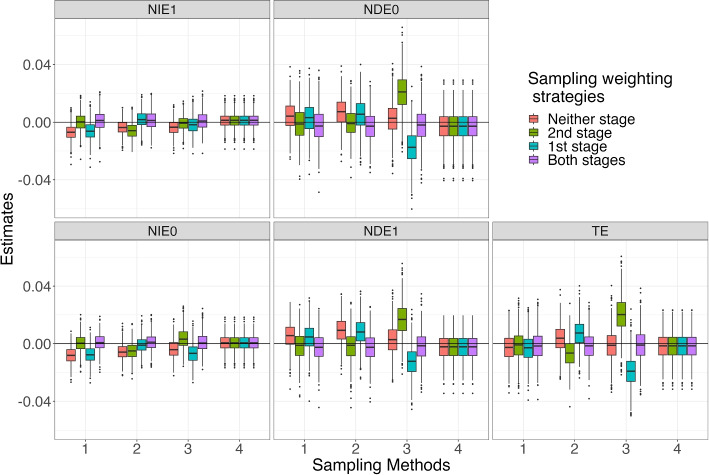
Distributions of Estimated Effects for Scenario 1. Note: Neither stage: no sampling weights in either stage; 1st stage: only use sampling weights to estimate mediation weights; 2nd stage: only use sampling weights in outcome model; Both stages: use sampling weights in both stages. Sampling Methods: 1. $S \sim U$, 2. $S \sim U + M$, 3. $S \sim U + A$, 4. $S \sim 1$

**Fig. 3:**
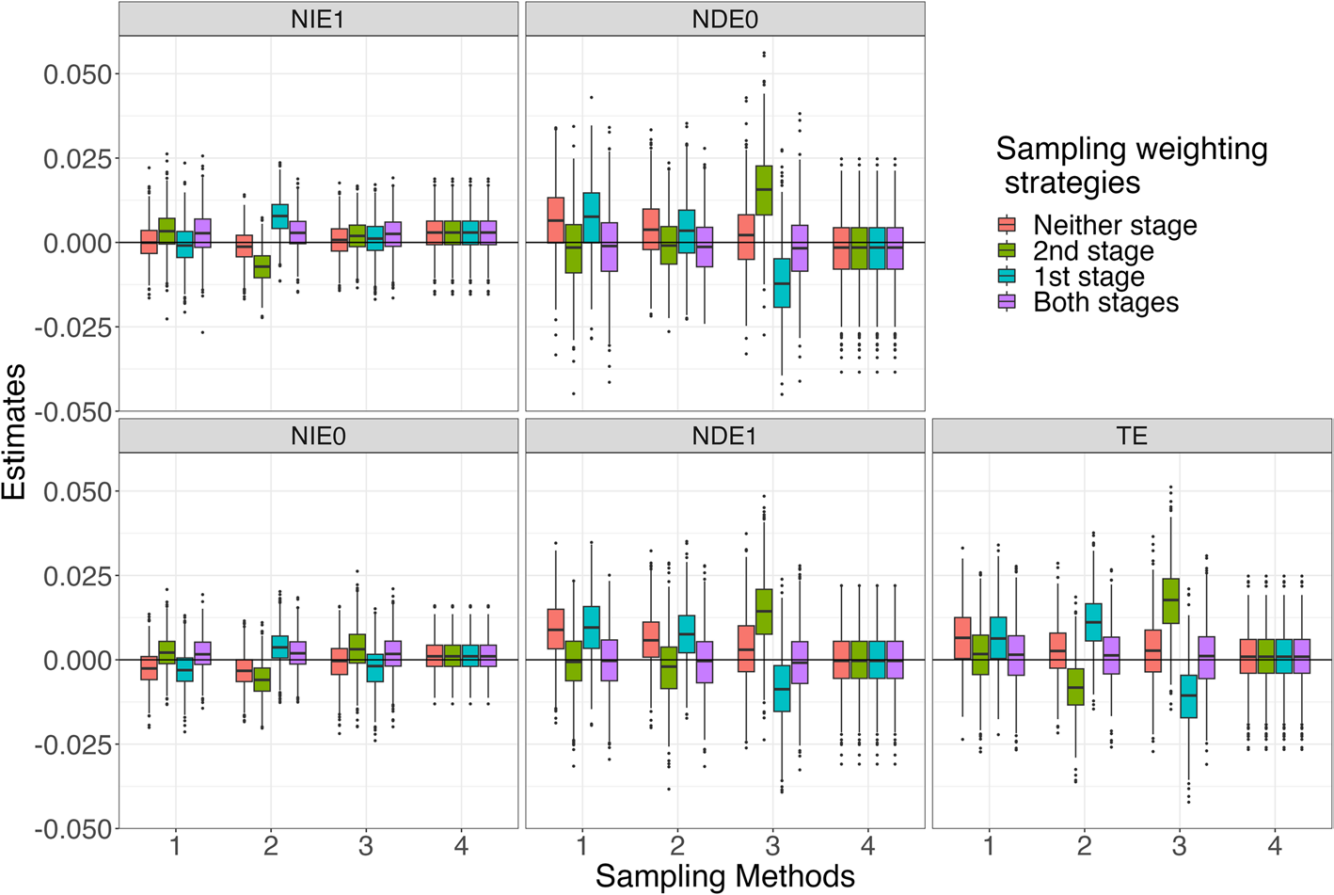
Distributions of Estimated Effects for Scenario 8. Note: Neither stage: no sampling weights in either stage; 1st stage: only use sampling weights to estimate mediation weights; 2nd stage: only use sampling weights in outcome model; Both stages: use sampling weights in both stages. Sampling Methods: 1. $S \sim U$, 2. $S \sim U + M$, 3. $S \sim U + A$, 4. $S \sim 1$

Figures 2 and 3 show the distributions of the differences between the 1,000 estimates and the true values for the effects of interest–*TE*, $NIE_1$, $NDE_0$, $NIE_0$, and $NDE_1$ for Scenarios 1 and 8, respectively. In each figure there is one panel for each effect. Each panel presents four sets of boxplots, one for each sampling method. Each set of boxplots contain four color-coded boxplots displaying the distributions of the differences between the estimated effects and the true population effect, for each of the four sampling weighting strategies (both stages – purple; first stage only – blue; second stage only – green; neither stage – red). The closer the center of the boxplots are to 0, the less bias in the estimates. The smaller the height of the box, the more precise the estimates are.

First we will consider results for Scenario 1 (see Fig. 2), focusing on TE estimation. Panel TE of Fig. 2 shows that, across all four sampling weighting strategies, using sampling weights in both stages yielded the smallest bias in the *TE* estimate (see purple boxplots). When sample selection depended on covariates and the mediator (Sampling Method 2) or covariates and the treatment (Sampling Method 3), using sampling weights in only the first or the second stage led to notably more biased *TE* estimates than weighting in both stages. For the *TE*, bias is small for all four sampling weighting strategies when sampling depends only on covariates (Sampling Method 1) and there is effectively no bias under simple random sampling (Sampling Method 4). We note that effect estimates were identical across weighting strategies for Sampling Method 4. The results for the direct and indirect effects are similar to those of the total effect, although biases tend to be smaller in magnitude for the indirect effects.

Turning to *TE* estimation for Scenario 8 (Fig. 3), we observe that using sampling weights in both stages yields smaller bias than does the other weighting strategies for Sampling Methods 1, 2, and 3. For Sampling Method 4, effect estimates were again identical across weighting strategies with very little bias. For the other, more complex, Sampling Methods 1, 2, and 3, the total effects are recovered comparably to that under simple random sampling. That is, they are all slightly positively biased but the magnitude of the bias is less when using sampling weights at both stages compared to using weights at only one or neither of the two stages. Consistent with the overall findings for the *TE* estimates, the distributions of the $NIE_1$, $NDE_0$, $NIE_0$, and $NDE_1$ estimates show that using sampling weights in both stages generally yielded the least biased results. Not using sampling weights in any part of the analysis resulted in inconsistent results across both sampling methods and estimators. For instance, for Sampling Method 3 (sampling depends on covariates and treatment), not using weights in either stage resulted in almost no bias for $NIE_1$ and $NIE_0$ yet positively biased results for *TE*, $NDE_0$, and $NDE_1$. For both Scenario 1 and 8, the variance of the estimates depends on the estimator but is mostly invariant to the sampling method or the sampling weighting strategy.

As shown in Appendix A, the distributions of effect estimates in Scenarios 2 to 7 had similar patterns as those in Scenarios 1 and 8. Specifically, using sampling weights at both stages led to the least biased estimates for 15 of 24 scenarios for *TE*, 17 for $NIE_1$, 15 for $NDE_0$, 18 for $NIE_0$, and 15 for $NDE_1$. Moreover, the pattern of bias across the sampling methods and weighting strategies are very similar to those for Scenarios 1 and 8. For example, the *TE* bias is positive and relatively large in magnitude for weighting only in the first stage and negative for weighting only in the second stage for Sampling Method 3 for every scenario. Similarly, as with Scenarios 1 and 8, bias and variance are somewhat larger for the direct effect estimates than the indirect effect estimates.

**Fig. 4:**
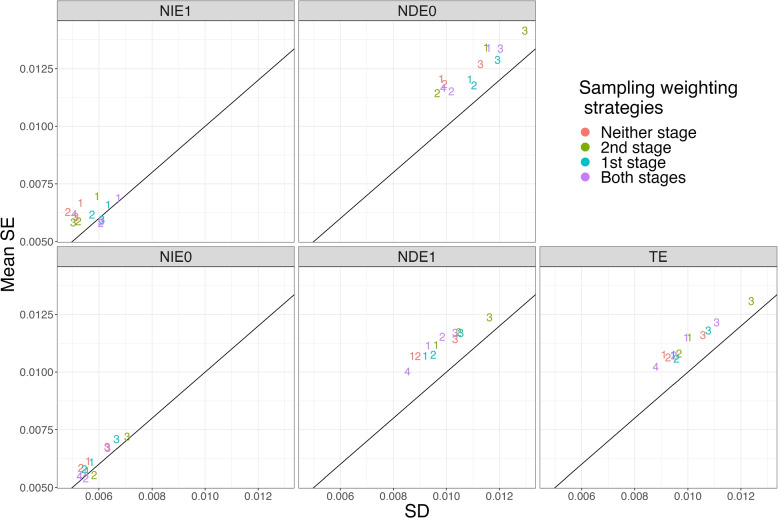
Standard Deviation (SD) vs Mean Standard Error (SE) of estimates for Scenario 1. Note: The plotting symbols 1 to 4 indicate the sampling methods: 1. $S \sim U$, 2. $S \sim U + M$, 3. $S \sim U + A$, 4. $S \sim 1$. The colors indicate the sampling weighting strategy: Neither stage: no sampling weights in either stage; 1st stage: only use sampling weights to estimate mediation weights; 2nd stage: only use sampling weights in outcome model; Both stages: use sampling weights in both stages

**Fig. 5:**
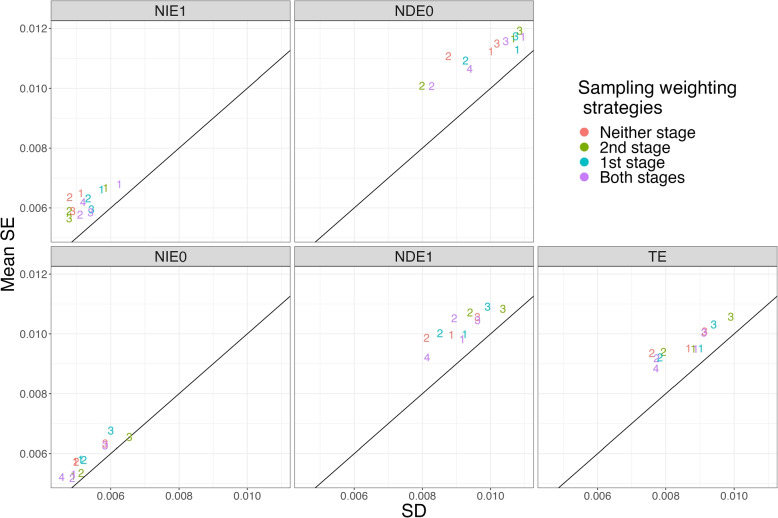
Standard Deviation (SD) vs Mean Standard Error (SE) of estimates for Scenario 8. Note: The plotting symbols 1 to 4 indicate the sampling methods: 1. $S \sim U$, 2. $S \sim U + M$, 3. $S \sim U + A$, 4. $S \sim 1$. The colors indicate the sampling weighting strategy: Neither stage: no sampling weights in either stage; 1st stage: only use sampling weights to estimate mediation weights; 2nd stage: only use sampling weights in outcome model; Both stages: use sampling weights in both stages

In addition to evaluating the bias and precision of the estimates under the different conditions, we evaluated the estimated standard errors of the estimates. Specifically, we compared the mean standard error estimates to the standard deviation of the estimates over the 1,000 replications. Figure 4 shows this comparison for the four sampling methods (as indicated by the numerical plotting symbol) by the four sampling weighting strategies (as indicated by the color of the plotting symbols) for Scenario 1. Figure 5 is the corresponding plot for Scenario 8, which shows similar patterns as Fig. 4. The results indicated that the uncertainty of the *TE* estimate was overestimated using the estimated standard error of *TE* in Scenario 1. The average standard errors were about 1.06 to 1.18 times the size of the standard deviation of the estimates. The standard errors for $NIE_1$, $NDE_0$, $NIE_0$, and $NDE_1$ were generally all overestimated as well. Some amount of overestimation of the standard errors is consistent with the literature. For example, [18] also found that sandwich standard errors overestimate total effect estimates calculated with estimated propensity scores. They explain that this is because sandwich standard errors do not account for correlation between the estimated means for the treatment and control groups resulting from estimation of the propensity scores. As noted by [19], estimation with estimated propensity scores is more efficient than estimation with the true propensity scores and sandwich estimators assume the weights are known.

For complete simulation study results, see Appendix A.

## Empirical study

We additionally considered a motivating empirical example that applies mediation analysis to health disparities research. Our specific focus is examining potential mediating pathways that explain smoking disparities among sexual minority (e.g., gay, lesbian, or bisexual) women, using data from the National Survey of Drug Use and Health (NSDUH). Specifically, lesbian, gay, and bisexual (LGB) women report higher rates of smoking than heterosexual women [20, 21]. We conceptualize sexual minority status as the exposure of interest, in that it gives rise to experiences of “minority stress”, namely excess social stressors experienced by individuals in a marginalized social group (e.g., LGB individuals). Manifestations of minority stress may include experiences of stigma, discrimination, bullying, and family rejection, among others. Smoking among LGB individuals has been theorized to reflect, in part, a coping strategy to minority stress experiences.

**Fig. 6:**
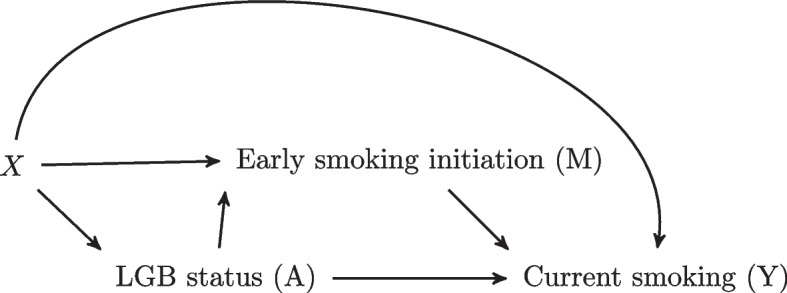
Graphical depiction of the effect of LGB status on adult smoking status as mediated by early smoking initiation

We apply mediation analysis to elucidate potential causal pathways that may give rise to these elevated rates of smoking. Our hypothesized mediator is early smoking initiation (i.e., prior to age 15), which is a strong risk factor for developing nicotine dependence. Specifically, we hypothesize that LGB women are more likely to begin smoking at an early age than heterosexual women, potentially in response to minority stressors. Resultantly, earlier smoking initiation among LGB women may contribute to higher rates of smoking among LGB women. In summary, the exposure is defined as sexual minority status (1=LGB women, 0=heterosexual women), the mediator is early smoking initiation (1=early initiation, 0=no early initiation), and the outcome is current smoking in adulthood (1=yes, 0=no). Baseline covariates include age, race/ethnicity, education level, household income, employment status, marital status, and urban vs. rural residence. Figure 6 illustrates our motivating example.

We applied the same four sampling weighting strategies as those in the simulation study-using NSDUH sampling weights in neither stage, in the first stage (mediation weights) only, in the second stage (outcome model) only, and in both stages. We fitted generalized boosted models (GBM) for the estimation of propensity scores and mediation weights. The *TE*, *NDE*, and *NIE* are compared across sampling weighting strategies.

Table 1 lists the effect estimates computed in this empirical study using the four sampling weighting strategies. Although we do not know the “true” effects to benchmark our estimates against, we find that these four strategies yield notably different estimates in this empirical context. For example, not using sampling weights yielded estimates for *TE*, $NDE_0$, and $NIE_0$ that were consistently larger in magnitude relative to using sampling weights at both stages. Estimates were also consistently larger in magnitude – relative to using sampling weights at both stages – when using weighting at the first stage only (with the exception of $NIE_1$). However, use of sampling weights in both stages yielded estimates that were consistently more variable (i.e., wider CIs) than other sampling weighting strategies. In this study, $NDE_0$ and $NIE_0$ were found not significantly different from 0. All the other effect estimates were significantly different from 0. However, in other analytic contexts, different sampling weighting strategies may yield different patterns of statistical significance for effect estimates, and hence differential inferences.

**Table 1:** Effect estimates for the empirical study

Sampling weighting	*TE*	$NDE_0$	$NIE_1$	$NDE_1$	$NIE_0$
Strategies	$(95\% CI)$	$(95\% CI)$	$(95\% CI)$	$(95\% CI)$	$(95\% CI)$
Neither stage	0.123	0.097	0.026	0.094	0.029
	(0.105, 0.141)	(0.080, 0.115)	(0.020, 0.031)	(0.076, 0.112)	(0.026, 0.031)
2nd Stage	0.103	0.081	0.022	0.079	0.024
	(0.073, 0.133)	(0.051, 0.111)	(0.014, 0.030)	(0.049, 0.110)	(0.021, 0.027)
1st Stage	0.123	0.072	0.051	0.113	0.010
	(0.105, 0.141)	(0.044, 0.100)	(0.030, 0.071)	(0.095, 0.132)	(0.004, 0.015)
Both Stages	0.103	0.049	0.054	0.099	0.004
	(0.073, 0.133)	(-0.001, 0.099)	(0.028, 0.081)	(0.067, 0.132)	(-0.007, 0.014)

## Discussion

Survey data collected with unequal probability sampling is often used for studying causal effects including decomposing total effects into direct and indirect effects through mediation analysis. A question that arises is how best to use sampling weights to estimate causal effects, including causal mediation effects, for the entire population. Through analytic results, we demonstrated that, under common assumptions for causal mediation analysis and assuming ignorability of sample selection given the sample design variables, it is possible to use sampling weights to consistently estimate the counterfactual population means necessary for estimating total effects, direct effects, and indirect effects. These results assume that a weighted estimation such as proposed by [14] will be used to estimate the effects. Our derivation shows that sampling weights should be used (1) when estimating the probability of treatment and (2) that the product of the sampling weight and the IPW or cross-world weights should be used when estimating the population means of the potential outcomes. That is, sampling weights should be used at both estimation stages.

Our analytic results show that using sampling weights at both stages is sufficient for consistent estimation under standard assumptions. They do not show it is necessary. It is possible that under some conditions unbiased estimates would be possible without weighting at both stages. However, the simulation study found that using sampling weights in both stages was less biased for all four sampling methods for all five effect estimates. Other weighting strategies yielded notably larger bias for one or more effects for one or more sampling methods. Also, the variance of the estimated effects was roughly the same across the four sampling weighting strategies for a given effect, sample design, and scenario. Thus, based on the analytic results and the simulation study, weighting in both stages appears to be the preferred approach. In the empirical example, we did find that weighting at both stages tended to yielded more variable estimates than using no weights or weighting only in the second stage. Thus, there may be a cost in terms of greater variance from using sampling weights in both stages as a tradeoff for less bias. This is an area that should be explored in future research studies. Another direction for future research is examining the inclusion of sampling weights in other estimators [22, 23] for causal mediation. These estimators do not require estimation of a propensity model for creating weights. Rather the question addressed would be whether to include the sampling weights at all.

The simulation study found that the commonly used sandwich standard error estimators tended to overestimate the standard error of the effect estimates across a range of simulated data conditions. The bias was smallest for total effects and occurred for all the sampling strategies and scenarios. Further research is needed to explore modifications to the sandwich estimators to correct the bias. For example, the sandwich estimator could be adjusted to account for the estimation of the probabilities of treatment used in creating the IPW and cross-world weights following the approach described in [24] when logistic regression is used to estimate the probability of treatment.

The analytic results are independent of the source of nonrandom sampling (unequal probability sample design, attrition, or nonresponse). A limitation of the simulation study is that it does not explore nonrandom sampling due to attrition or nonresponse; it is not guaranteed that the relative performance of the estimators would be the same for nonresponse or attrition as they were in the simulation study. Under some conditions, such as interactions between exposure and covariates related to sample inclusion, use of the weights in the estimate of the conditional probabilities will be necessary to avoid bias. As such, we conclude that using the weights in both stages of the analysis is a valuable strategy for ensuring the results are robust to bias, regardless of the source of nonrandom sampling. Additionally, our estimand of interest was the conditional probability of receiving treatment among the population, not among the sample. Furthermore, we note that prior authors [7–9] also considered the use of the sampling weights as a covariate to estimate propensity scores. This same approach might be used as an alternative in the context of mediation analysis. Again, the key is that using the weights in both estimation stages provides robustness for causal mediation with nonrandom samples.

## Conclusion

The combination of our analytic and simulation study results create a compelling case for using the sampling weights in both stages of the causal mediation analysis when using weighted estimators. However, there are some limitations to our study. First our simulation study only considers relatively large samples of 9, 000 participants. Given that weighting can reduce the precision of estimates, the cost of weighting at both stages to avoid possible bias might be greater for small samples. That could result in one of the other sampling strategies yielding less biased estimated effects when the sample sizes are smaller. Second, the simulation study only considered dichotomous variables for the outcome, mediator, and confounders. Use of dichotomous confounders and mediators constrains the IPW and cross-world weights to a small number of possible values. This might have limited the impact of weighting in the first stage on the variability of the final estimates. Third, the simulation study only considered the use of logistic regression to estimate the probability of treatment. Again results might differ when more flexible models such as GBM are used to estimate the probability of treatment.

### Supplementary Information

Supplementary Material 1.

## Data Availability

The NSDUH data are publicly available and are also included in the twangMediation R package, which is available on CRAN. The R code for the NSDUH analysis are included in the supplementary materials. The R code for running the simulation is available upon request.

## Abbreviations

ATT: Average treatment effect among the treated
CI: Confidence interval
ESS: Effective sample size
GBM: Generalized boosted model
GPS: Generalized propensity score
IPW: Inverse probability weight
LGB: Lesbian, gay, or bisexual
MSE: Mean squared error
NDE: Natural direct effect
NIE: Natural indirect effect
NSDUH: National Survey of Drug Use and Health
PS: Propensity score
RMSE: Root mean squared error
SD: Standard deviation
SE: Standard error
TE: Total effect
