# Supplementary material for: Sampling weighting strategies in causal mediation analysis
Source: BMC Med Res Methodol. 2024 Jun 15;24:133. doi: 10.1186/s12874-024-02262-x (PMC11179247; doi:10.1186/s12874-024-02262-x)
Supplement: Supplementary file 1 — Supplementary Material 1. [file 12874_2024_2262_MOESM1_ESM.pdf]

## Supplementary Material

# Appendix A

This is the supplementary material for the paper "Sampling Weighting Strategies in Causal Mediation Analysis".

### Distributions of effect estimates

Figure S1: Distributions of Estimated Effects for Scenario 2

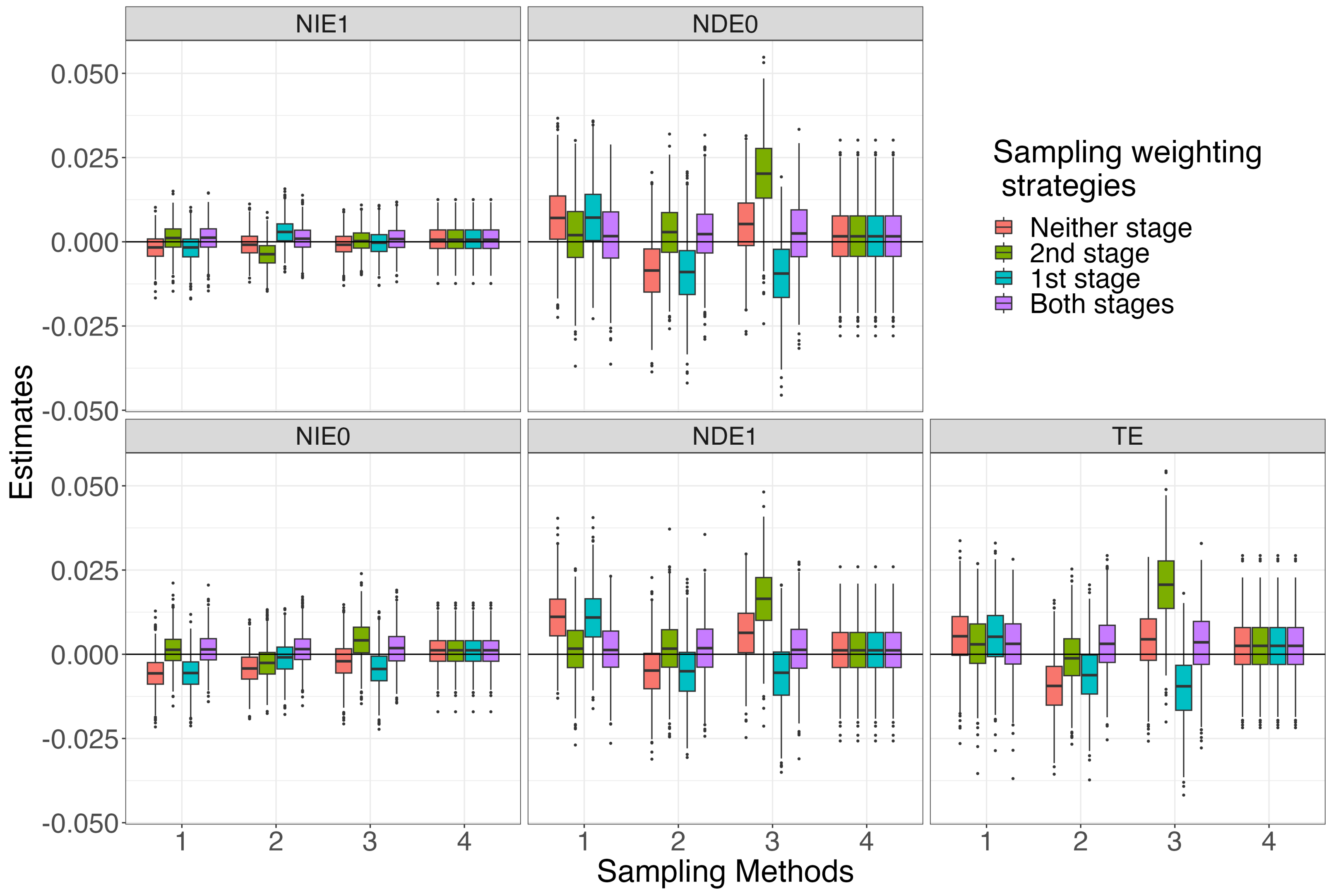


Note: Neither stage: no sampling weights in either stage; 1st stage: only use sampling weights to estimate mediation weights; 2nd stage: only use sampling weights in outcome model; Both stages: use sampling weights in both stages.

Sampling Methods: 1. $S \sim U$, 2. $S \sim U + M$, 3. $S \sim U + A$, 4. $S \sim 1$.

Figure S2: Distributions of Estimated Effects for Scenario 3

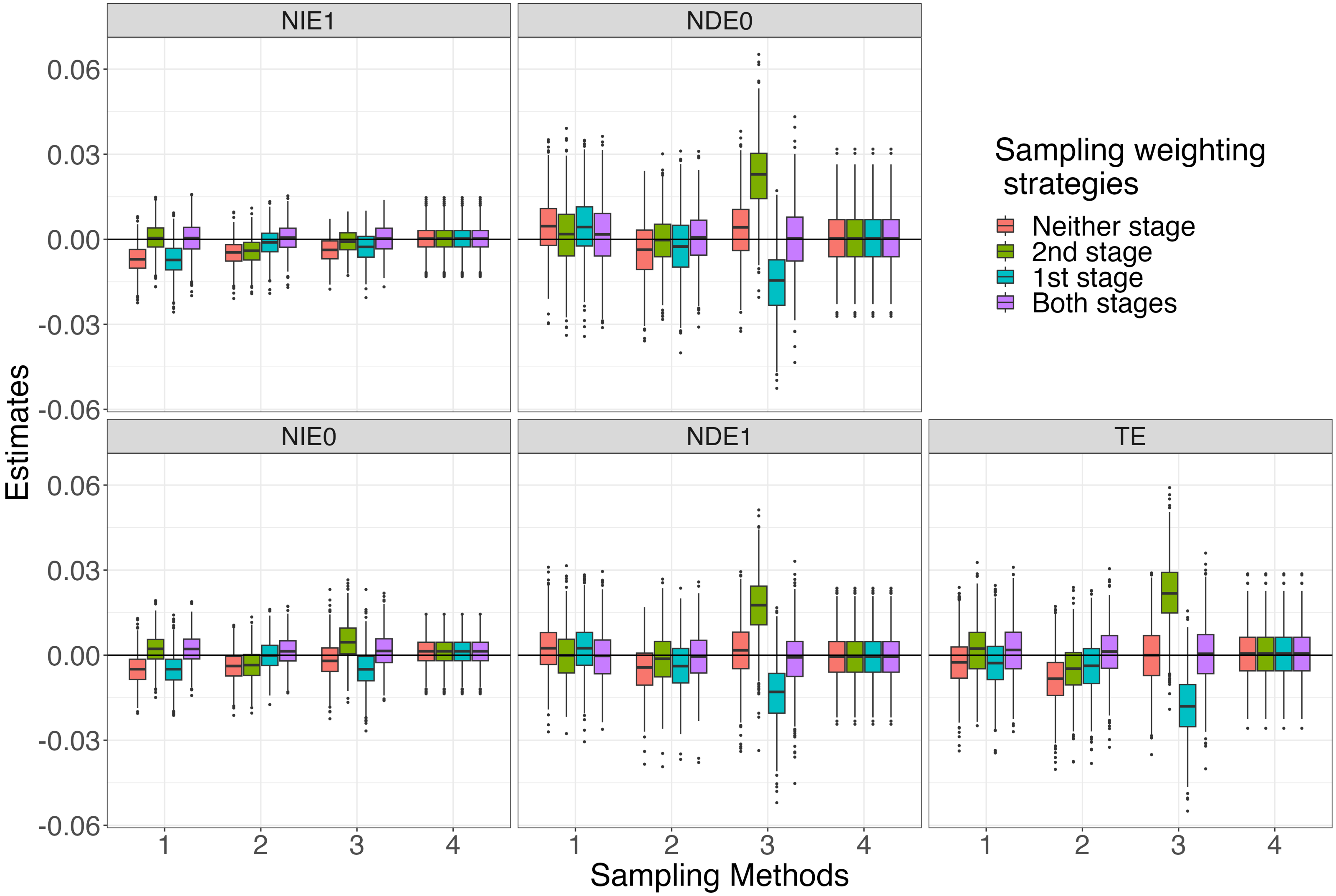


Note: Neither stage: no sampling weights in either stage; 1st stage: only use sampling weights to estimate mediation weights; 2nd stage: only use sampling weights in outcome model; Both stages: use sampling weights in both stages.

Sampling Methods: 1. $S \sim U$, 2. $S \sim U + M$, 3. $S \sim U + A$, 4. $S \sim 1$.

Figure S3: Distributions of Estimated Effects for Scenario 4

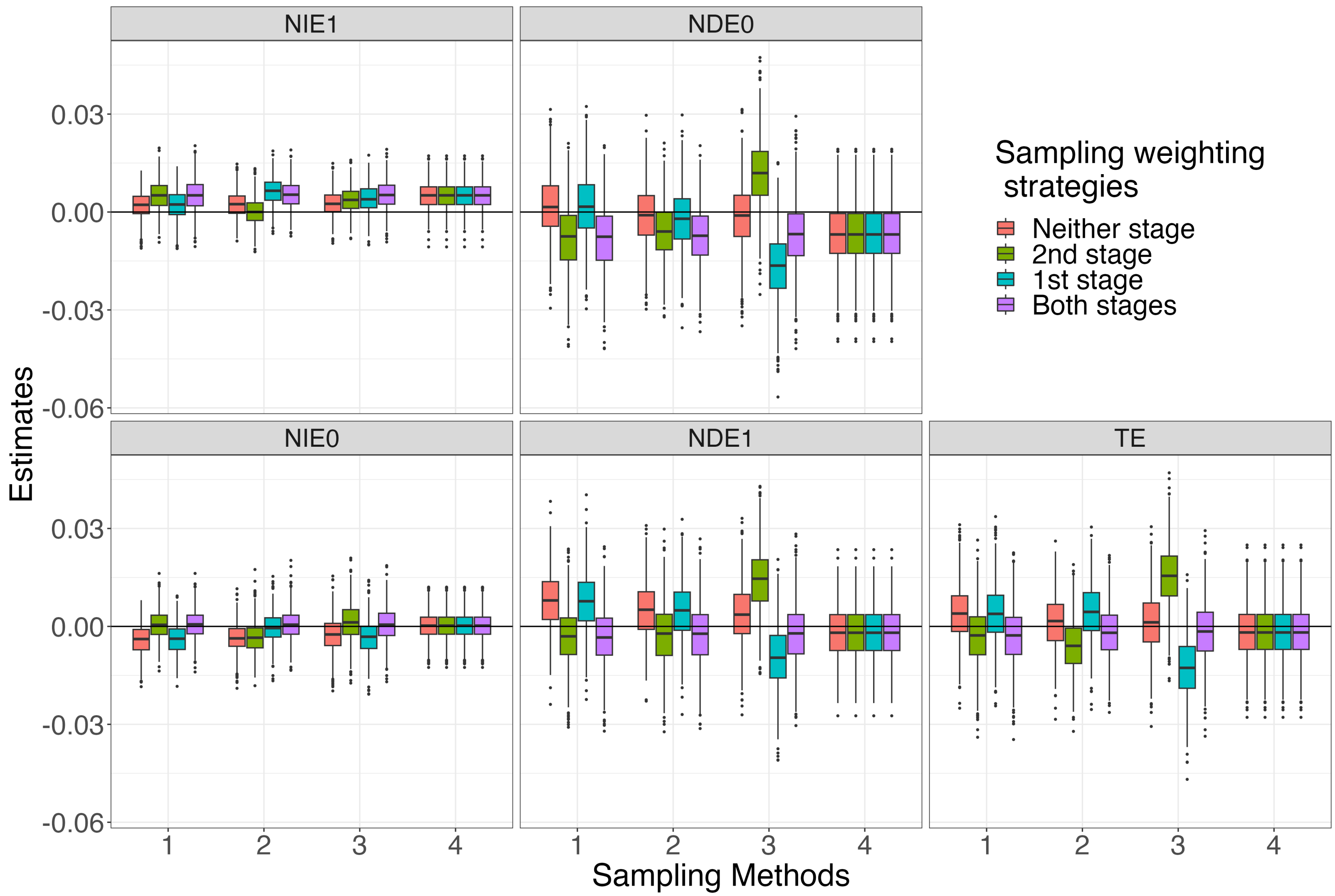


Note: Neither stage: no sampling weights in either stage; 1st stage: only use sampling weights to estimate mediation weights; 2nd stage: only use sampling weights in outcome model; Both stages: use sampling weights in both stages.

Sampling Methods: 1. $S \sim U$, 2. $S \sim U + M$, 3. $S \sim U + A$, 4. $S \sim 1$.

Figure S4: Distributions of Estimated Effects for Scenario 5

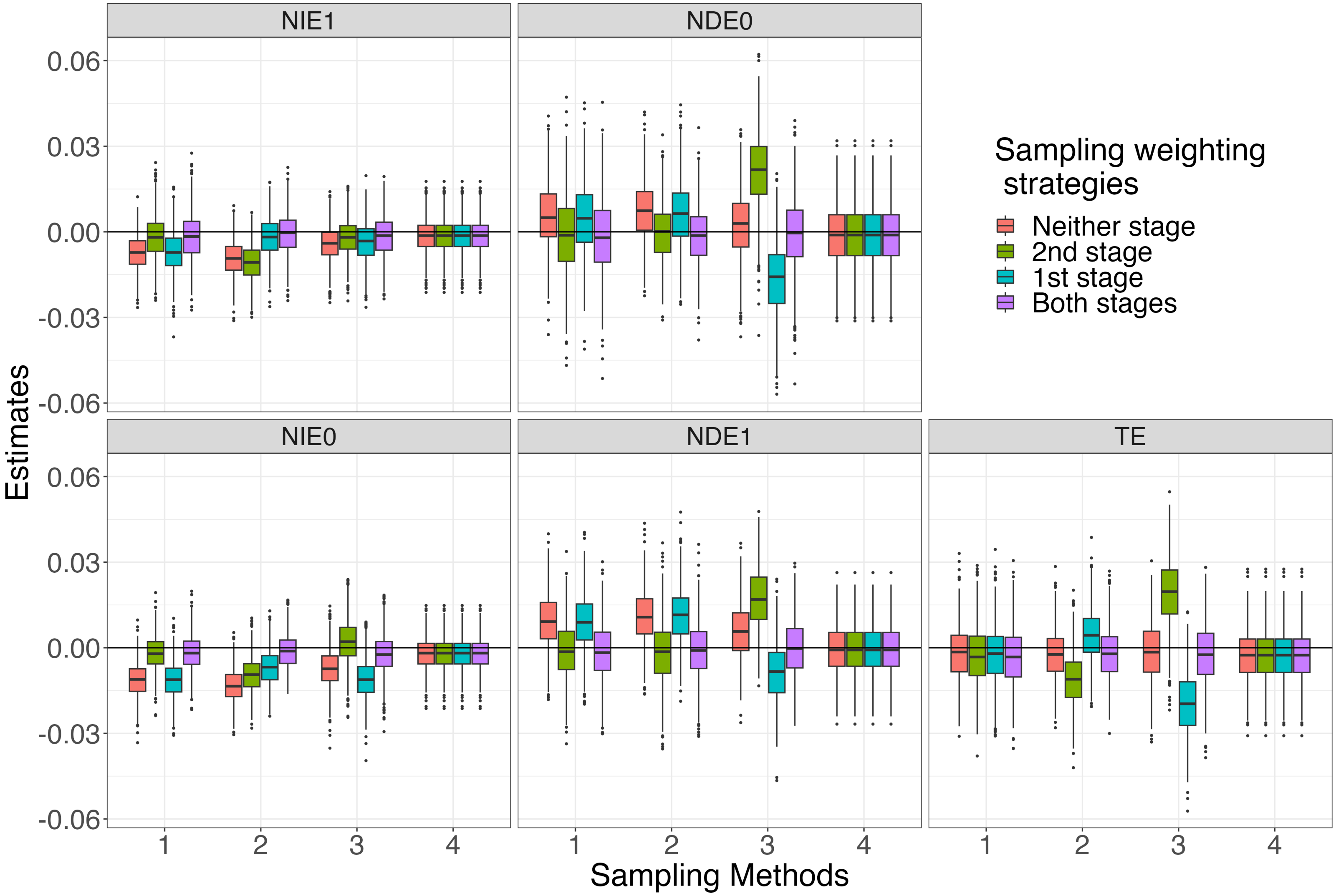


Note: Neither stage: no sampling weights in either stage; 1st stage: only use sampling weights to estimate mediation weights; 2nd stage: only use sampling weights in outcome model; Both stages: use sampling weights in both stages.

Sampling Methods: 1. $S \sim U$, 2. $S \sim U + M$, 3. $S \sim U + A$, 4. $S \sim 1$.

Figure S5: Distributions of Estimated Effects for Scenario 6

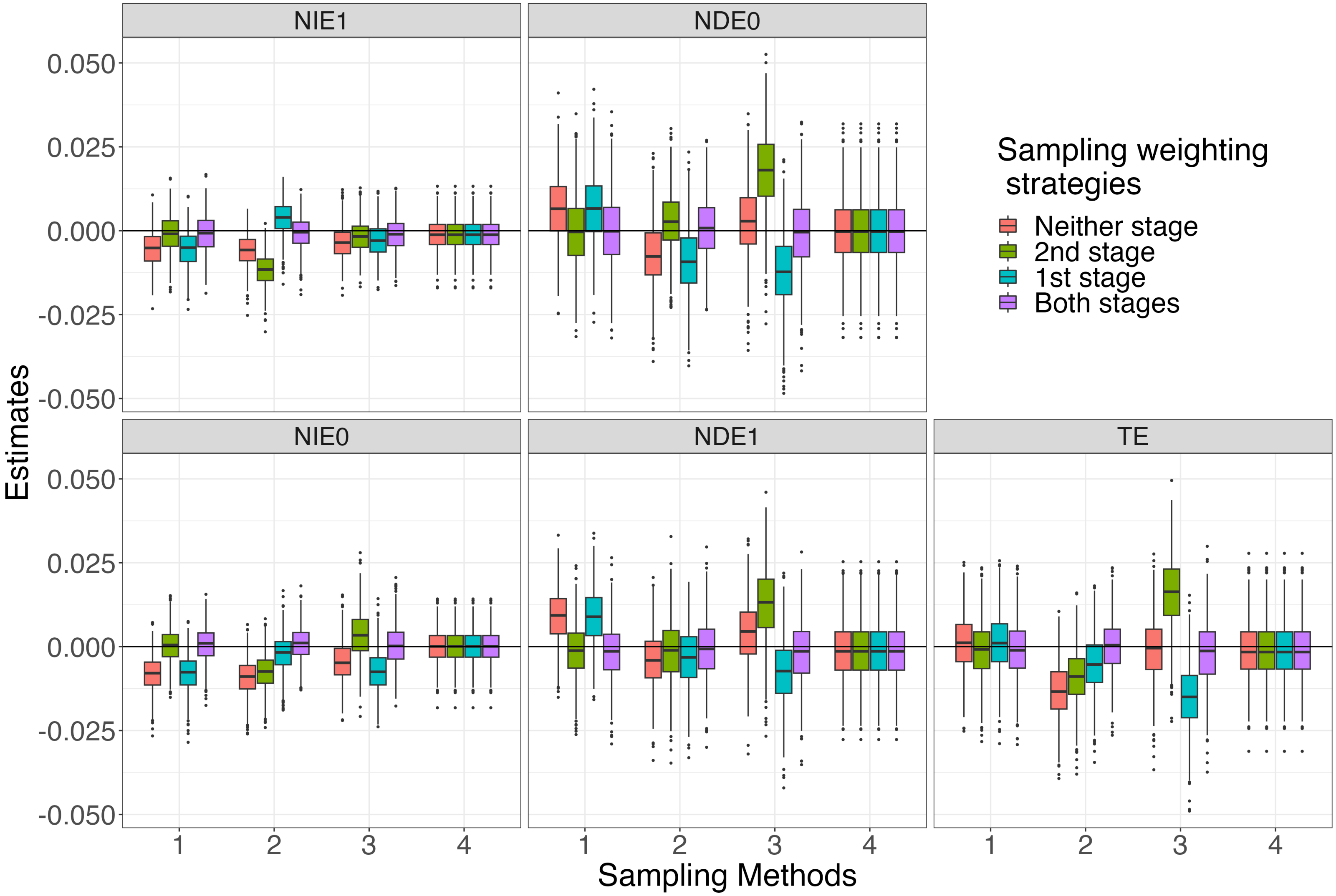


Note: Neither stage: no sampling weights in either stage; 1st stage: only use sampling weights to estimate mediation weights; 2nd stage: only use sampling weights in outcome model; Both stages: use sampling weights in both stages.

Sampling Methods: 1. $S \sim U$, 2. $S \sim U + M$, 3. $S \sim U + A$, 4. $S \sim 1$.

Figure S6: Distributions of Estimated Effects for Scenario 7

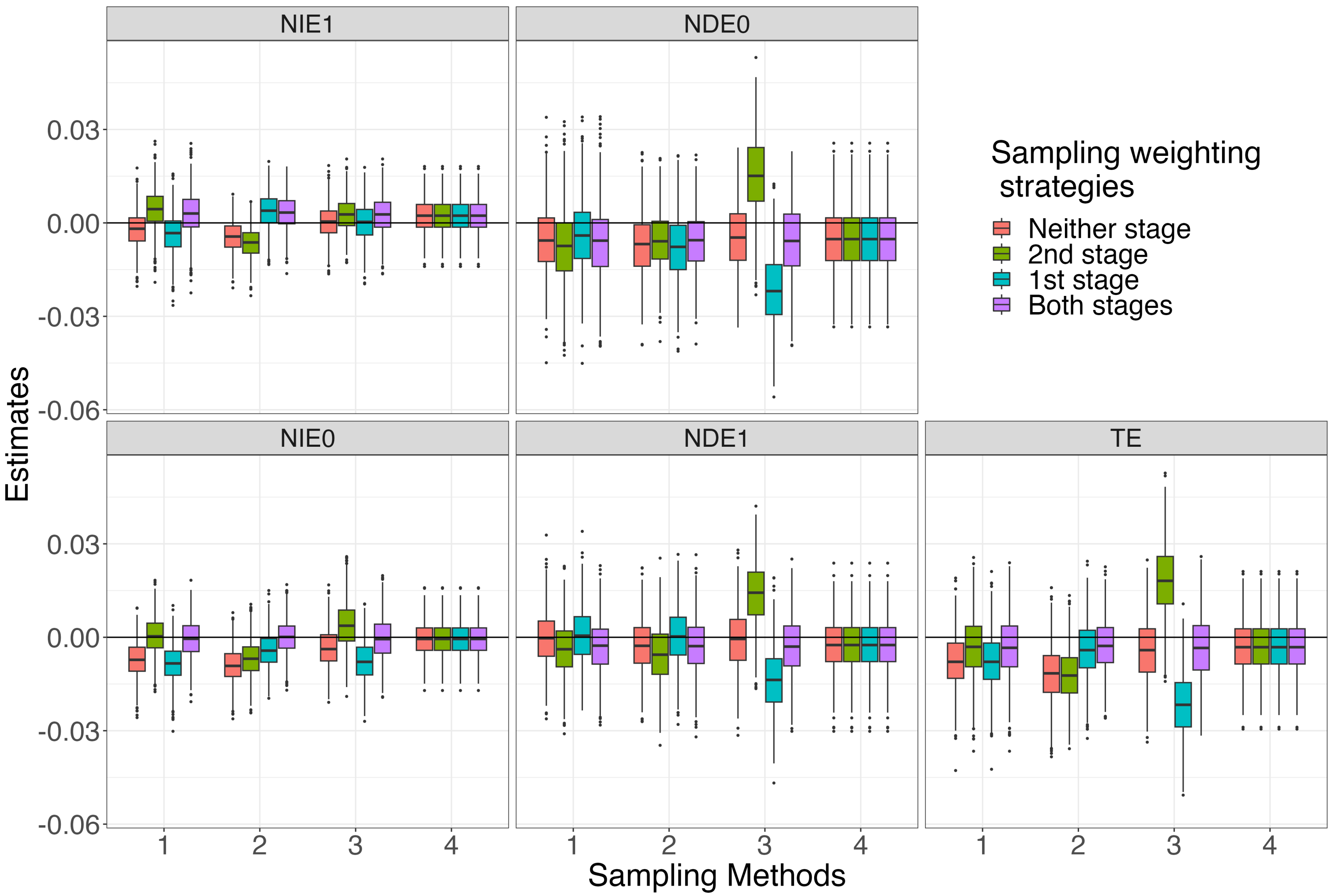


Note: Neither stage: no sampling weights in either stage; 1st stage: only use sampling weights to estimate mediation weights; 2nd stage: only use sampling weights in outcome model; Both stages: use sampling weights in both stages.

Sampling Methods: 1. $S \sim U$, 2. $S \sim U + M$, 3. $S \sim U + A$, 4. $S \sim 1$.

**Comparison of Standard Deviation and Mean Standard Error of Effect Estimates over 1,000 Replications.**

Figure S7: Standard Deviation (SD) vs Mean Standard Error (SE) of estimates for Scenario 2

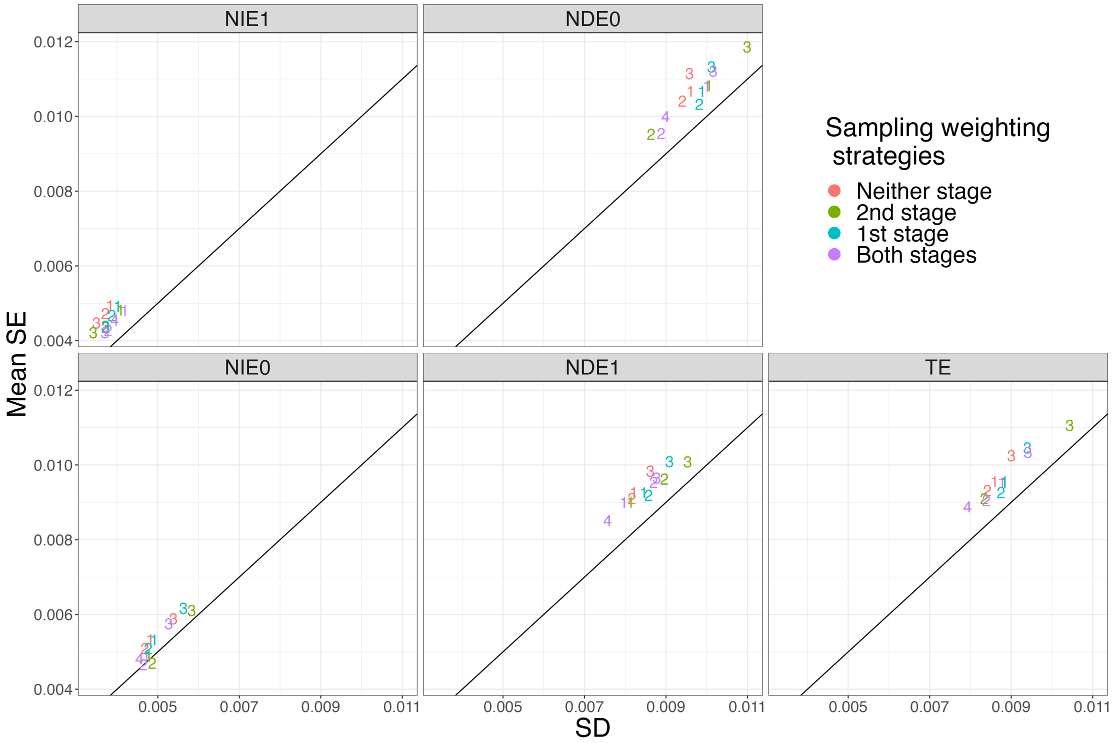


Note: Neither stage: no sampling weights in either stage; 1st stage: only use sampling weights to estimate mediation weights; 2nd stage: only use sampling weights in outcome model; Both stages: use sampling weights in both stages.

Figure S8: Standard Deviation (SD) vs Mean Standard Error (SE) of estimates for Scenario 3

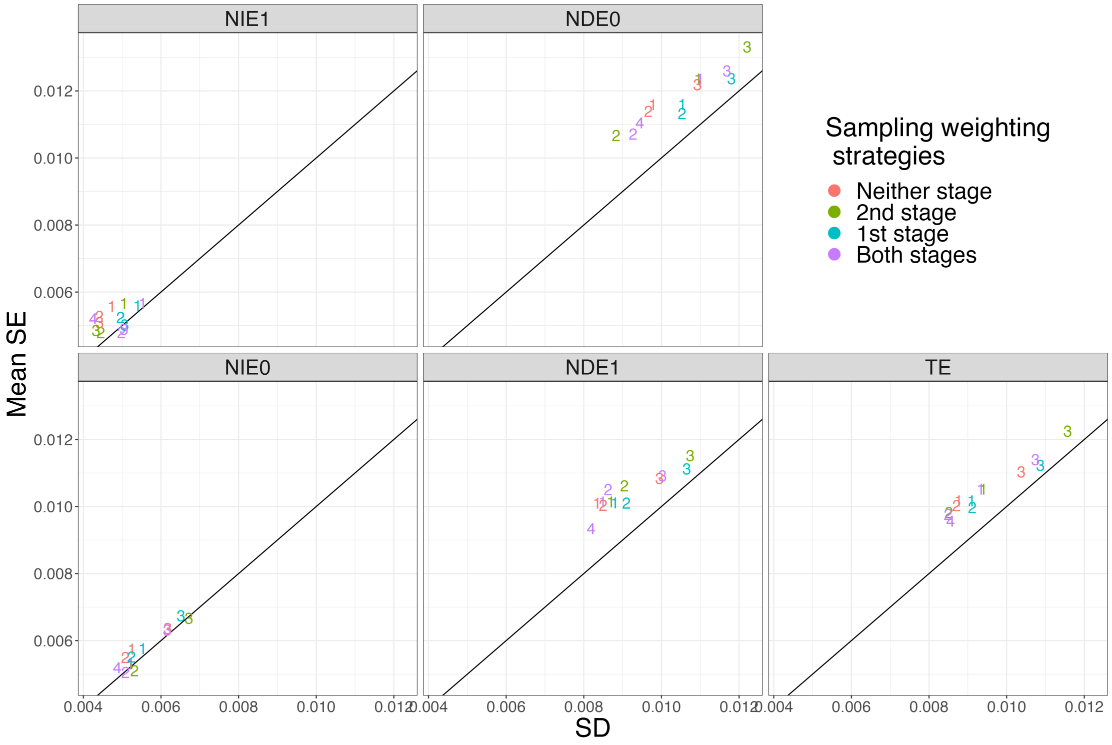


Note: Neither stage: no sampling weights in either stage; 1st stage: only use sampling weights to estimate mediation weights; 2nd stage: only use sampling weights in outcome model; Both stages: use sampling weights in both stages.

Figure S9: Standard Deviation (SD) vs Mean Standard Error (SE) of estimates for Scenario 4

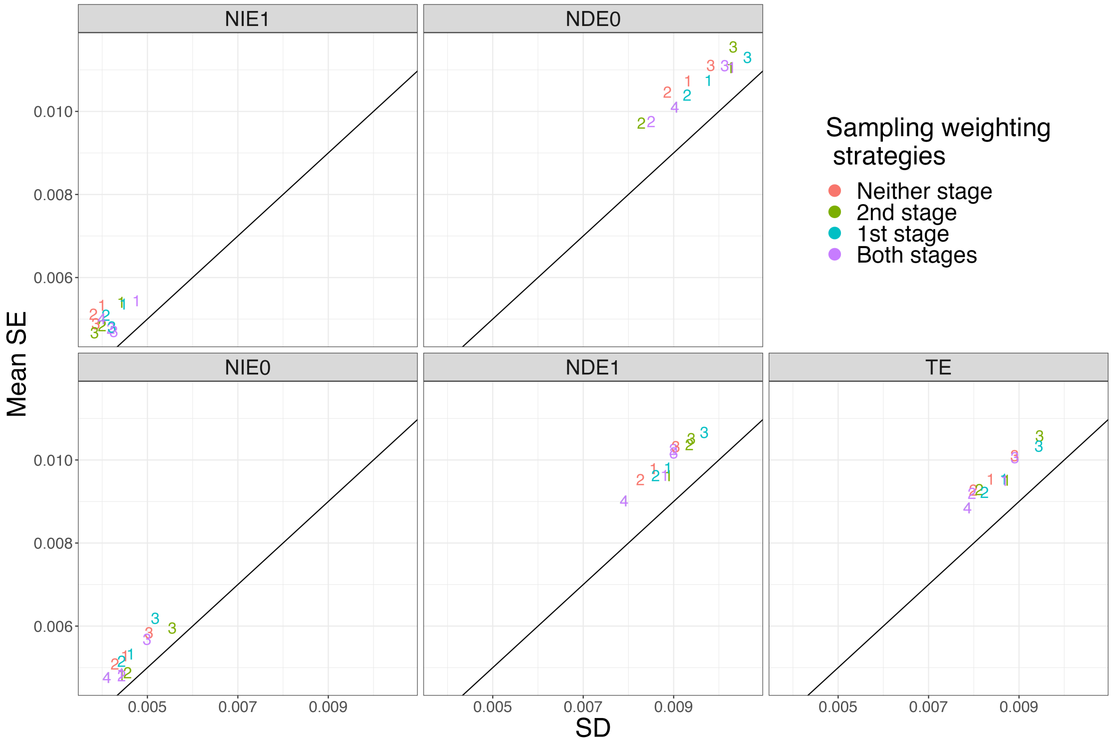


Note: Neither stage: no sampling weights in either stage; 1st stage: only use sampling weights to estimate mediation weights; 2nd stage: only use sampling weights in outcome model; Both stages: use sampling weights in both stages.

Figure S10: Standard Deviation (SD) vs Mean Standard Error (SE) of estimates for Scenario 5

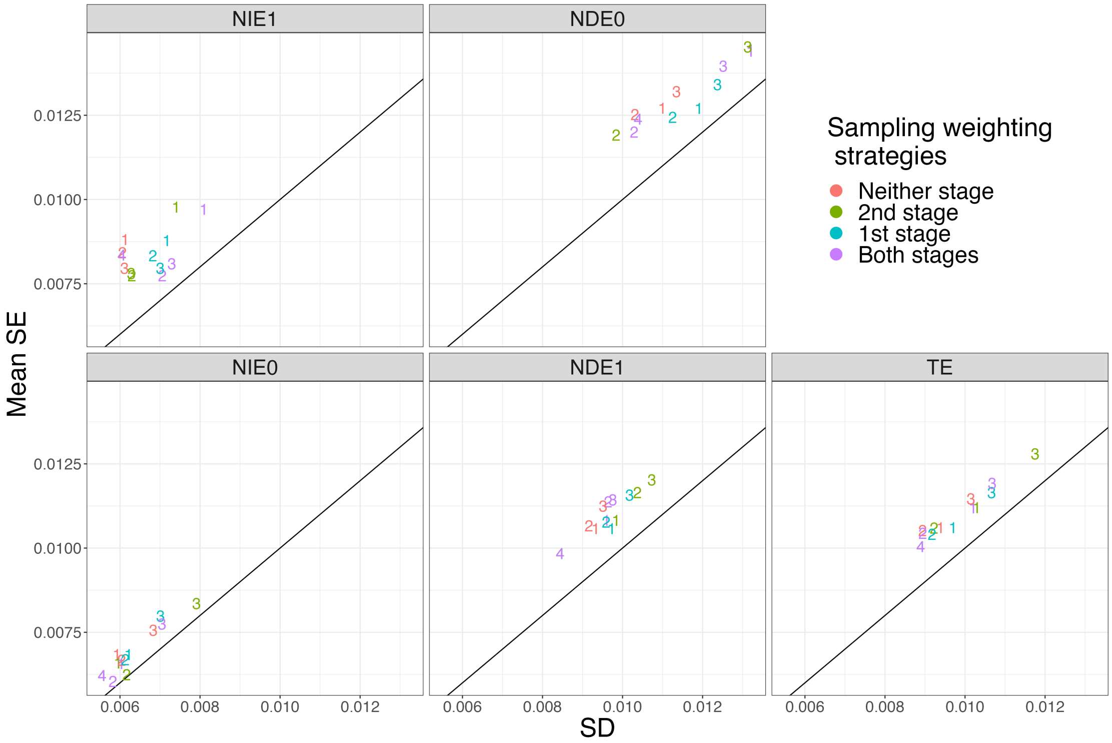


Note: Neither stage: no sampling weights in either stage; 1st stage: only use sampling weights to estimate mediation weights; 2nd stage: only use sampling weights in outcome model; Both stages: use sampling weights in both stages.

Figure S11: Standard Deviation (SD) vs Mean Standard Error (SE) of estimates for Scenario 6

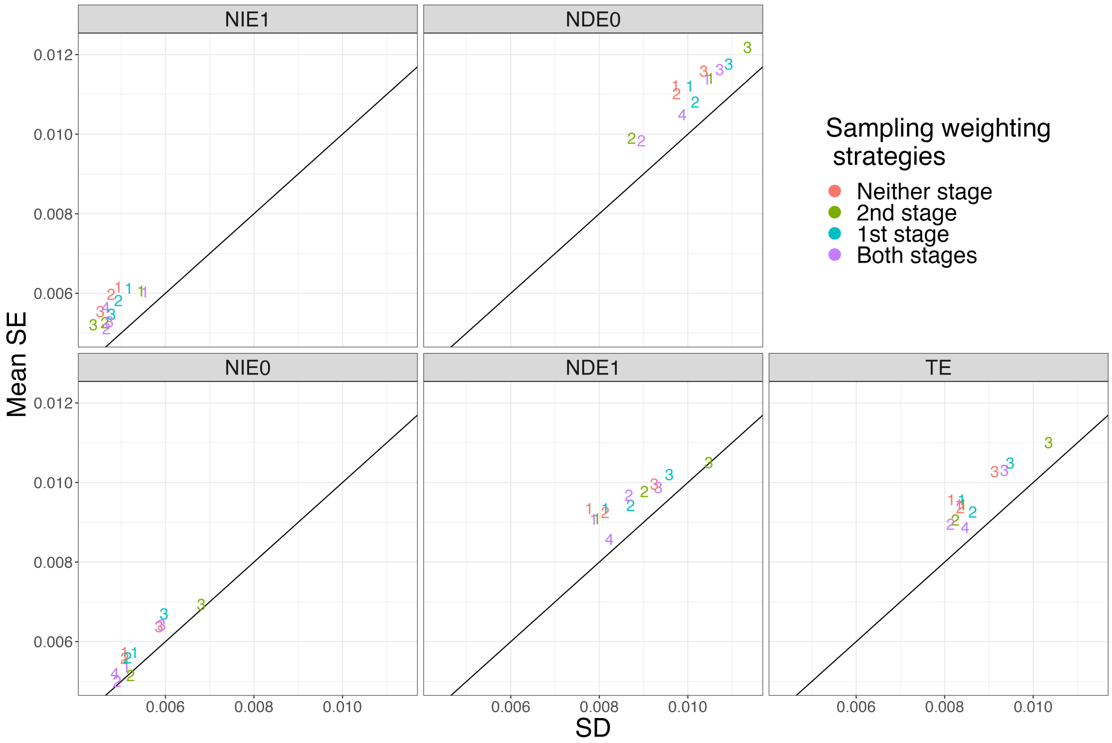


Note: Neither stage: no sampling weights in either stage; 1st stage: only use sampling weights to estimate mediation weights; 2nd stage: only use sampling weights in outcome model; Both stages: use sampling weights in both stages.

Figure S12: Standard Deviation (SD) vs Mean Standard Error (SE) of estimates for Scenario 7

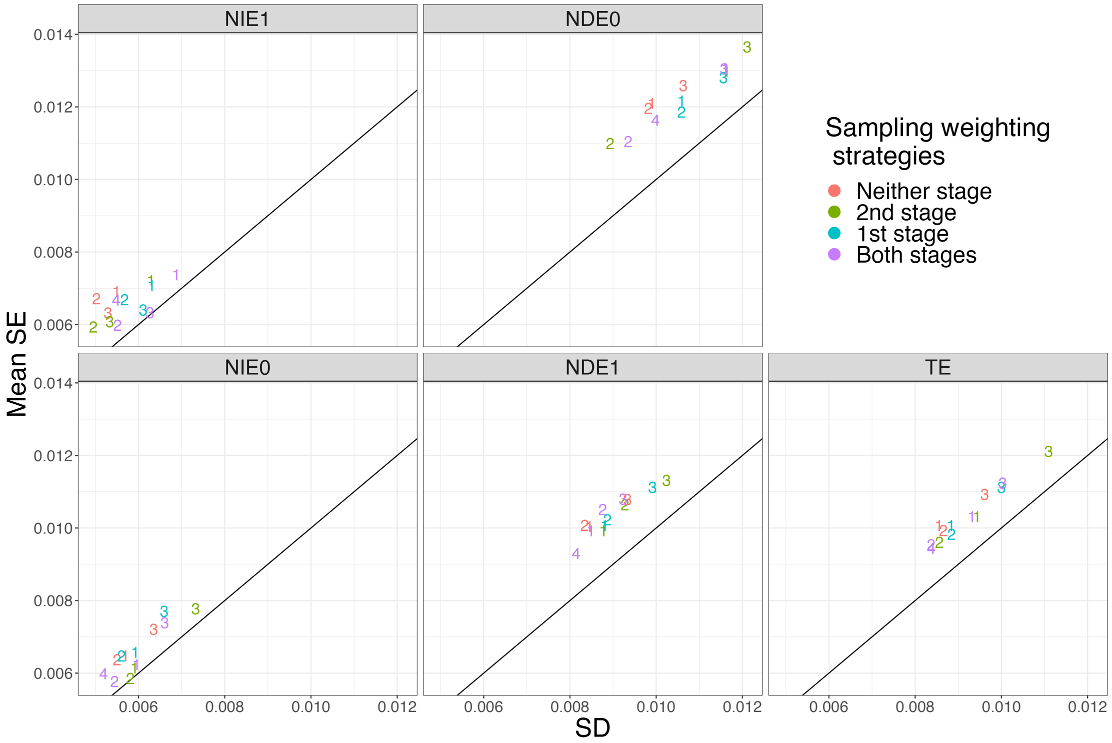


Note: Neither stage: no sampling weights in either stage; 1st stage: only use sampling weights to estimate mediation weights; 2nd stage: only use sampling weights in outcome model; Both stages: use sampling weights in both stages.

## Appendix B

**Data analysis code for the empirical study**

```
## Empirical Study
library(twang)
library(twangMediation)
load("./input_sim/NSDUH_female.rda")

# "34" means weighting strategies 3 and 4.
ps_model_emp12 <- glm(formula = lgb_flag ~ age + race + educ + income + employ,
                      data = NSDUH_female, family = "binomial")
ps_model_emp34 <- glm(formula = lgb_flag ~ age + race + educ + income + employ,
    data = NSDUH_female, family = "binomial", weights = NSDUH_female$NSDUHwt)
NSDUH_female$ps12 <- predict(ps_model_emp12,
                                NSDUH_female[,
                                        c("age", "race", "educ", "income", "employ")],
                                        type = "response")

NSDUH_female$ps34 <- predict(ps_model_emp34,
                                NSDUH_female[,
                                 c("age", "race", "educ", "income", "employ")],
                                 type = "response")

# inverse probability weights (total effect weights)
NSDUH_female$tew12 <- ifelse(NSDUH_female$lgb_flag ==1, 1/NSDUH_female$ps12,
                                1/(1-NSDUH_female$ps12))
NSDUH_female$tew34 <- ifelse(NSDUH_female$lgb_flag ==1, 1/NSDUH_female$ps34,
                                1/(1-NSDUH_female$ps34))

# standardize sampling weights
NSDUH_female$spw_s <- NSDUH_female$NSDUHwt/sum(NSDUH_female$NSDUHwt)
```

```
cig_med_wt1 <- wgtmed(cig15 ~ age + race + educ + income + employ,
                     a_treatment="lgb_flag",
                     y_outcome="cigmon",
                     data = NSDUH_female, method = "logistic",
                     total_effect_wts = NSDUH_female$tew12)

cig_med_wt2 <- wgtmed(cig15 ~ age + race + educ + income + employ,
                      a_treatment="lgb_flag",
                      y_outcome="cigmon",
                      data = NSDUH_female, method = "logistic",
                      total_effect_wts = NSDUH_female$tew12,
                      sampw = NSDUH_female$spw_s)

cig_med_wt3<- wgtmed(cig15 ~ age + race + educ + income + employ,
                      a_treatment="lgb_flag",
                      y_outcome="cigmon",
                      data = NSDUH_female, method = "logistic",
                      total_effect_wts = NSDUH_female$tew34)

cig_med_wt4<- wgtmed(cig15 ~ age + race + educ + income + employ,
                     a_treatment="lgb_flag",
                     y_outcome="cigmon",
                     data = NSDUH_female, method = "logistic",
                     total_effect_wts = NSDUH_female$tew34,
                     sampw = NSDUH_female$spw_s)

## Use GBM instead.
#no sampw
TEps_wt12 <- ps(lgb_flag ~ age + race + educ + income + employ,
```

```
              data=NSDUH_female, verbose=F, n.trees=10000, n.keep = 5,
              estimand = "ATE")
# sampw
TEps_wt34 <- ps(lgb_flag ~ age + race + educ + income + employ,
              data=NSDUH_female, verbose=F, n.trees=10000, n.keep = 5,
              estimand = "ATE",
              sampw = NSDUH_female$NSDUHwt)
# sw strategy 1
cig_med_wt1 <- wgtmed(cig15 ~ age + race + educ + income + employ,
                     a_treatment="lgb_flag",
                     y_outcome="cigmon",
                     data=NSDUH_female,
                     method="ps",
                     total_effect_ps=TEps_wt12,
                     total_effect_stop_rule="ks.mean",
                     ps_version="gbm",
                     ps_n.trees=10000,
                     ps_n.keep = 5,
                     ps_stop.method="ks.mean")
# sw strategy 2
cig_med_wt2 <- wgtmed(cig15 ~ age + race + educ + income + employ,
                     a_treatment="lgb_flag",
                     y_outcome="cigmon",
                     data=NSDUH_female,
                     method="ps",
                     total_effect_ps=TEps_wt12,
                     total_effect_stop_rule="ks.mean",
                     ps_version="gbm",
                     ps_n.trees=10000,
                     ps_n.keep = 5,
                     ps_stop.method="ks.mean",
```

```
                     sampw = NSDUH_female$NSDUHwt)
# sw strategy 3
cig_med_wt3 <- wgtmed(cig15 ~ age + race + educ + income + employ,
                     a_treatment="lgb_flag",
                     y_outcome="cigmon",
                     data=NSDUH_female,
                     method="ps",
                     total_effect_ps=TEps_wt34,
                     total_effect_stop_rule="ks.mean",
                     ps_version="gbm",
                     ps_n.trees=10000,
                     ps_n.keep = 5,
                     ps_stop.method="ks.mean")
# sw strategy 4
cig_med_wt4 <- wgtmed(cig15 ~ age + race + educ + income + employ,
                     a_treatment="lgb_flag",
                     y_outcome="cigmon",
                     data=NSDUH_female,
                     method="ps",
                     total_effect_ps=TEps_wt34,
                     total_effect_stop_rule="ks.mean",
                     ps_version="gbm",
                     ps_n.trees=10000,
                     ps_n.keep = 5,
                     ps_stop.method="ks.mean",
                     sampw = NSDUH_female$NSDUHwt)
# output effect estimates and statistics
summary(cig_med_wt1)
summary(cig_med_wt2)
summary(cig_med_wt3)
summary(cig_med_wt4)
```

### Simulation code

```
start_time1 <- Sys.time()
##### rap the scripts together to run Rscript command in HPC
## Unlike R CMD BATCH, Rscript does not save results in memory, but is significantly
## faster in Compute. The reason is not sure.
#### Script 1: re_sim_haoyu.R

# simulation project
# 12/02/2021

# First generate the simulated population data based on the determined parameters.
# Check distributions. Then decide whether or not to adjust parameters.
setwd("/home/tuo70113/simulation_project/output_sim")

library(dplyr)
library(stats)
library(readr)

set.seed(12)
# set population size
N <- 90000
stratum <- c(rep(1,30000), rep(2, 30000), rep(3,30000))
# covariate distribution
# V3: make all covariates dichotomous
U1 <- c(rbinom(N/3, 1, 0.3), rbinom(N/3, 1, 0.5), rbinom(N/3, 1, 0.7))
U2 <- c(rbinom(N/3, 1, 0.35), rbinom(N/3, 1, 0.4), rbinom(N/3, 1, 0.6))
U3 <- c(rbinom(N/3, 1, 0.25), rbinom(N/3, 1, 0.6), rbinom(N/3, 1, 0.75))
X1 <- c(rbinom(N/3, 1, 0.43), rbinom(N/3, 1, 0.57), rbinom(N/3, 1, 0.92))
X2 <- c(rbinom(N/3, 1, 0.24), rbinom(N/3, 1, 0.42), rbinom(N/3, 1, 0.87))
X3 <- c(rbinom(N/3, 1, 0.2), rbinom(N/3, 1, 0.4), rbinom(N/3, 1, 0.8))
cov <- cbind(U1, U2, U3, X1, X2, X3)

#### 8 population data scenarios (model combinations,
#not accounting for sampling selection):
#1 treatment model, 2 mediator models, and 4 outcome models.
```

```
# new solution for data generation by Dan:
# a = alpha1 * U1 + alpha2 * U2 + alpha3 * U3 + alpha4 * X1 + alpha5 * X2 + alpha6 * X3
# b = beta1 * U1 + beta2 * U2 + beta3 * U3 + beta4 * X1 + beta5 * X2 + beta6 * X3
# with interaction: d = delta*X2; without interaction: d = a constant
# d = delta * X2

### Notes:
#scenario 1: A ~ C, M ~ A + C, Y ~ A + M + C
#scenario 2: A ~ C, M ~ A + C, Y ~ A + M + A*M + C
#scenario 3: A ~ C, M ~ A + C, Y ~ A + M + C + A * one of C
#scenario 4: A ~ C, M ~ A + C, Y ~ A + M + C + M * one of C
#scenario 5: A ~ C, M ~ A + C + A * one of C, Y ~ A + M + C
#scenario 6: A ~ C, M ~ A + C + A * one of C, Y ~ A + M + A*M + C
#scenario 7: A ~ C, M ~ A + C + A * one of C, Y ~ A + M + C + A * one of C
#scenario 8: A ~ C, M ~ A + C + A * one of C, Y ~ A + M + C + M * one of C

## treatment model
alpha <- c(0.68, -0.25, -0.2, 0.2, 0.4, -0.63, 0.3)
beta <- c(0.8, -0.23, 0.2, 0.39, 0.42, -0.51, 0.76)
# d must be smaller than the negative of min(a - b) = -2.49
d_noint <- -1.52
d_int <- -1.52 - 0.5*X2 #(X2: the interacted covariate)
a <- alpha[1] + rowSums(sweep(cov, MARGIN = 2, alpha[2:length(alpha)], FUN = "*"))
b <- beta[1] + rowSums(sweep(cov, MARGIN = 2, beta[2:length(beta)], FUN = "*"))

# check constraints
all(a < b)
all((a-b)>d_noint)
all((a-b)>d_int)

p_treat <- 1/(1 + exp(-a))

#replace: treat_noint<- ifelse(p_treat_noint>=0.5, yes = 1, no = 0)
treat <- rbinom(N, 1, p_treat)
```

```
# treat_all stores all treatment values for 24 scenarios
treat_all <- data.frame(matrix(NA, nrow = N, ncol = 8))
for (scenario in 1:8){
        treat_all[,scenario] <- treat

}

## mediator models
p_mediator <- data.frame(matrix(NA, nrow = N, ncol = 8))
mediator_all<- data.frame(matrix(NA, nrow = N, ncol = 8))
for (scenario in 1:8){
        if (scenario %in% 1:4){# no interaction of A*C in Model M, and no A given M
                p_mediator[,scenario] <-
                        (exp(-a) - exp(-b))/
                        (treat* (exp(-b-d_noint) - exp(-b)) + (1-treat)* (exp(-a) - exp(d_noint-a)))
        }else{# within interaction of A*C in Model M, and M*C in A given M
                p_mediator[,scenario] <-
                        (exp(-a) - exp(-b))/
                        (treat* (exp(-b-d_int) - exp(-b)) + (1-treat)* (exp(-a) - exp(d_int-a)))
        }
}

# mediator_all contains the binary mediator values for all scenarios
#replace: mediator_all <- ifelse(p_mediator>=0.5, yes = 1, no = 0)
for (scenario in 1:8){
        mediator_all[,scenario] <- rbinom(N, 1, p_mediator[,scenario])
}

## outcome model
p_outcome <- data.frame(matrix(NA, nrow = N, ncol = 4))
outcome_all <- data.frame(matrix(NA, nrow = N, ncol = 4))
for (scenario in 1:8){
        if (scenario %in% c(1,5)){#outcome model 1: Y ~ A + M + C
                p_outcome[,scenario] <- 1/(1+exp(-(-0.61 +0.62*treat_all[,scenario]
                + 2.0*mediator_all[,scenario]
                                                  -1.42*cov[,1] -1.08*cov[,2] -0.77*cov[,3]
```

```
                                          -1.08*cov[,4] +0.51*cov[,5] +0.36*cov[,6])))

        }else if (scenario %in% c(2,6)){#outcome model 2: Y ~ A + M + C + A*M
                p_outcome[,scenario] <- 1/(1+exp(-(-0.61 +0.65*treat_all[,scenario]
                + 1.7*mediator_all[,scenario]
                                          -1.42*cov[,1] -1.07*cov[,2] -0.77*cov[,3]
                                          -1.07*cov[,4] -0.28*cov[,5] +0.36*cov[,6]
                                          -1.0*treat_all[,scenario]*mediator_all[,scenario])))

        }else if (scenario %in% c(3,7)){#outcome model 3: Y ~ A + M + C + A* (one of C)
                p_outcome[,scenario] <- 1/(1+exp(-(-0.61 +0.64*treat_all[,scenario]
                + 1.8*mediator_all[,scenario]
                                          -1.42*cov[,1] -1.08*cov[,2] -0.78*cov[,3]
                                          -1.08*cov[,4] +0.50*cov[,5] +0.36*cov[,6]
                                          -1.2*treat_all[,scenario]*cov[,5])))

        }else{#outcome model 4: Y ~ A + M + C + M * (one of C)
                p_outcome[,scenario] <- 1/(1+exp(-(-0.71 +0.61*treat_all[,scenario] +1.63*mediator_all[
                                          -1.39*cov[,1] -1.08*cov[,2] -0.75*cov[,3]
                                          -1.09*cov[,4] +0.60*cov[,5] +0.35*cov[,6]
                                          -1.45*mediator_all[,scenario]*cov[,5])))
        }
}

# outcome_all contains the binary outcome values for all scenarios
# replaced: outcome_all <- ifelse(p_outcome>= 0.5, yes = 1, no = 0)
for (scenario in 1:8 ){
        outcome_all[,scenario] <- rbinom(N, 1, p_outcome[,scenario])
}

### Assemble scenarios (put together correlated variables in each scenario)
data_pop <- vector("list", 8)
for (scenario in 1:8){
        data_pop[[scenario]]<- data.frame(matrix(NA, nrow = N, ncol = 9))
        colnames(data_pop[[scenario]])<-
                c("treat", "mediator", "outcome", "U1", "U2", "U3", "X1", "X2", "X3")
```

```
}
for (scenario in 1:8){
        data_pop[[scenario]][,1]<- treat_all[,scenario]
        data_pop[[scenario]][,4:9]<- cov # all covariates are the same across model scenarios
        data_pop[[scenario]][,2]<- mediator_all[,scenario]
        data_pop[[scenario]][,3]<- outcome_all[,scenario]
}

##Check distributions of covariates, treatment, mediator, and outcome.
for (scenario in 1:8){
        cat("Scenario:", scenario, "\n")
        for (i in c(1:8)){
                print(colnames(data_pop[[scenario]])[i])
                print(table(data_pop[[scenario]][i]))
        }
}

# correlation between continuous covariates
#cor.test(data_pop[[1]]$U2, data_pop[[1]]$U3, method = "spearman")
#cor.test(data_pop[[1]]$U2, data_pop[[1]]$X3, method = "spearman")
#cor.test(data_pop[[1]]$U3, data_pop[[1]]$X3, method = "spearman")
# or
#cor(data_pop[[1]][,c("U2", "U3", "X3")], method = "spearman")

# Chi-square test between binary covariates
cross_tab <- vector("list", length = 3)
cross_tab[[1]] <- xtabs(~U1+U2, data = data_pop[[1]])
cross_tab[[2]] <- xtabs(~U1+U3, data = data_pop[[1]])
cross_tab[[3]] <- xtabs(~U1+X1, data = data_pop[[1]])
cross_tab[[4]] <- xtabs(~U1+X2, data = data_pop[[1]])
cross_tab[[5]] <- xtabs(~U1+X3, data = data_pop[[1]])
cross_tab[[6]] <- xtabs(~U2+U3, data = data_pop[[1]])
cross_tab[[7]] <- xtabs(~U2+X1, data = data_pop[[1]])
cross_tab[[8]] <- xtabs(~U2+X2, data = data_pop[[1]])
cross_tab[[9]] <- xtabs(~U2+X3, data = data_pop[[1]])
cross_tab[[10]] <- xtabs(~U3+X1, data = data_pop[[1]])
```

```
cross_tab[[11]] <- xtabs(~U3+X2, data = data_pop[[1]])
cross_tab[[12]] <- xtabs(~U3+X3, data = data_pop[[1]])
cross_tab[[13]] <- xtabs(~X1+X2, data = data_pop[[1]])
cross_tab[[14]] <- xtabs(~X1+X3, data = data_pop[[1]])
cross_tab[[15]] <- xtabs(~U2+X3, data = data_pop[[1]])

for (i in 1:length(cross_tab)){
        print(cross_tab[[i]])
        print(summary(cross_tab[[i]]))
}

#crosstabs of treat - mediator, mediator- outcome, treat-outcome
for (scenario in 1 : 8){
        cat("Scenario:", scenario, "\n")
        cross_tmo <- vector("list", length = 3)
        cross_tmo[[1]] <-  xtabs(~treat+mediator, data = data_pop[[scenario]])
        cross_tmo[[2]] <-  xtabs(~mediator + outcome, data = data_pop[[scenario]])
        cross_tmo[[3]] <-  xtabs(~treat+outcome, data = data_pop[[scenario]])
        for (i in 1:3){
                print(cross_tmo[[i]])
                print(summary(cross_tmo[[i]]))
        }
        cat("\n")

}

# scenario 1 raw data
head(data_pop[[1]])

#### Script 2: counterfactuals.R
# True effects based on counterfactuals' comparison

## m0 and m1
p_m0 <- data.frame(matrix(NA, nrow = N, ncol = 8))
m0_all<- data.frame(matrix(NA, nrow = N, ncol = 8))
```

```
p_m1 <- data.frame(matrix(NA, nrow = N, ncol = 8))
m1_all<- data.frame(matrix(NA, nrow = N, ncol = 8))

set.seed(7612248)
#m0: treat ==0
for (scenario in 1:8){
        if (scenario %in% 1:4){# no interaction of A*C in Model M, and no A given M
                p_m0[,scenario] <- (exp(-b)-exp(-a))/(exp(d_noint-a)-exp(-a))
        }else{# within interaction of A*C in Model M, and M*C in A given M
                p_m0[,scenario] <- (exp(-b)-exp(-a))/(exp(d_int-a)-exp(-a))
        }
}

for (scenario in 1:8){
        m0_all[,scenario] <- rbinom(N, 1, p_m0[,scenario])
}

#m1:treat ==1
for (scenario in 1:8){
        if (scenario %in% 1:4){# no interaction of A*C in Model M, and no A given M
                p_m1[,scenario] <- (exp(-b)-exp(-a))/(exp(-b)-exp(-b-d_noint))
        }else{# within interaction of A*C in Model M, and M*C in A given M
                p_m1[,scenario] <- (exp(-b)-exp(-a)) / (exp(-b)-exp(-b-d_int))
        }
}
for (scenario in 1:8){
        m1_all[,scenario] <- rbinom(N, 1, p_m1[,scenario])
}

## outcome models
# Y0M0
p_y0m0 <- data.frame(matrix(NA, nrow = N, ncol = 4))
y0m0_all <- data.frame(matrix(NA, nrow = N, ncol = 4))
for (scenario in 1:8){
        if (scenario %in% c(1,5)){#outcome model 1: Y ~ A + M + C
                p_y0m0[,scenario] <- 1/(1+exp(-(-0.61 +0.62*0 +2.0*m0_all[,scenario]
```

```
                                        -1.42*cov[,1] -1.08*cov[,2] -0.77*cov[,3]
                                        -1.08*cov[,4] +0.51*cov[,5] +0.36*cov[,6])))

        }else if (scenario %in% c(2,6)){#outcome model 2: Y ~ A + M + C + A*M
                p_y0m0[,scenario] <- 1/(1+exp(-(-0.61 +0.65*0 +1.7*m0_all[,scenario]
                                        -1.42*cov[,1] -1.07*cov[,2] -0.77*cov[,3]
                                        -1.07*cov[,4] -0.28*cov[,5] +0.36*cov[,6]
                                        -1.0*0*m0_all[,scenario])))

        }else if (scenario %in% c(3,7)){#outcome model 3: Y ~ A + M + C + A* (one of C)
                p_y0m0[,scenario] <- 1/(1+exp(-(-0.61 +0.64*0 +1.8*m0_all[,scenario]
                                        -1.42*cov[,1] -1.08*cov[,2] -0.78*cov[,3]
                                        -1.08*cov[,4] +0.50*cov[,5] +0.36*cov[,6]
                                        -1.2*0*cov[,5])))

        }else{#outcome model 4: Y ~ A + M + C + M * (one of C)
                p_y0m0[,scenario] <- 1/(1+exp(-(-0.71 +0.61*0 +1.63*m0_all[,scenario]
                                        -1.39*cov[,1] -1.08*cov[,2] -0.75*cov[,3]
                                        -1.09*cov[,4] +0.60*cov[,5] +0.35*cov[,6]
                                        -1.45*m0_all[,scenario]*cov[,5])))
        }
}

# y0m0_all contains the binary outcome value of potential Y0M0 in all scenarios
for (scenario in 1:8 ){
        y0m0_all[,scenario] <- rbinom(N, 1, p_y0m0[,scenario])
}

# Y1M1
p_y1m1 <- data.frame(matrix(NA, nrow = N, ncol = 4))
y1m1_all <- data.frame(matrix(NA, nrow = N, ncol = 4))
for (scenario in 1:8){
        if (scenario %in% c(1,5)){#outcome model 1: Y ~ A + M + C
                p_y1m1[,scenario] <- 1/(1+exp(-(-0.61 +0.62*1 +2.0*m1_all[,scenario]
                                        -1.42*cov[,1] -1.08*cov[,2] -0.77*cov[,3]
                                        -1.08*cov[,4] +0.51*cov[,5] +0.36*cov[,6])))
```

```
        }else if (scenario %in% c(2,6)){#outcome model 2: Y ~ A + M + C + A*M
                p_y1m1[,scenario] <- 1/(1+exp(-(-0.61 +0.65*1 +1.7*m1_all[,scenario]
                                                -1.42*cov[,1] -1.07*cov[,2] -0.77*cov[,3]
                                                -1.07*cov[,4] -0.28*cov[,5] +0.36*cov[,6]
                                                -1.0*1*m1_all[,scenario])))

        }else if (scenario %in% c(3,7)){#outcome model 3: Y ~ A + M + C + A* (one of C)
                p_y1m1[,scenario] <- 1/(1+exp(-(-0.61 +0.64*1 +1.8*m1_all[,scenario]
                                                -1.42*cov[,1] -1.08*cov[,2] -0.78*cov[,3]
                                                -1.08*cov[,4] +0.50*cov[,5] +0.36*cov[,6]
                                                -1.2*1*cov[,5])))

        }else{#outcome model 4: Y ~ A + M + C + M * (one of C)
                p_y1m1[,scenario] <- 1/(1+exp(-(-0.71 +0.61*1 +1.63*m1_all[,scenario]
                                                -1.39*cov[,1] -1.08*cov[,2] -0.75*cov[,3]
                                                -1.09*cov[,4] +0.60*cov[,5] +0.35*cov[,6]
                                                -1.45*m1_all[,scenario]*cov[,5])))
        }
}

# y1m1_all contains the binary outcome value of potential Y0M0 in all scenarios
for (scenario in 1:8 ){
        y1m1_all[,scenario] <- rbinom(N, 1, p_y1m1[,scenario])
}

# Y1M0
p_y1m0 <- data.frame(matrix(NA, nrow = N, ncol = 4))
y1m0_all <- data.frame(matrix(NA, nrow = N, ncol = 4))
for (scenario in 1:8){
        if (scenario %in% c(1,5)){#outcome model 1: Y ~ A + M + C
                p_y1m0[,scenario] <- 1/(1+exp(-(-0.61 +0.62*1 +2.0*m0_all[,scenario]
                                                -1.42*cov[,1] -1.08*cov[,2] -0.77*cov[,3]
                                                -1.08*cov[,4] +0.51*cov[,5] +0.36*cov[,6])))

        }else if (scenario %in% c(2,6)){#outcome model 2: Y ~ A + M + C + A*M
```

```
            p_y1m0[,scenario] <- 1/(1+exp(-(-0.61 +0.65*1 +1.7*m0_all[,scenario]
                                            -1.42*cov[,1] -1.07*cov[,2] -0.77*cov[,3]
                                            -1.07*cov[,4] -0.28*cov[,5] +0.36*cov[,6]
                                            -1.0*1*m0_all[,scenario])))

        }else if (scenario %in% c(3,7)){#outcome model 3: Y ~ A + M + C + A* (one of C)
            p_y1m0[,scenario] <- 1/(1+exp(-(-0.61 +0.64*1 +1.8*m0_all[,scenario]
                                            -1.42*cov[,1] -1.08*cov[,2] -0.78*cov[,3]
                                            -1.08*cov[,4] +0.50*cov[,5] +0.36*cov[,6]
                                            -1.2*1*cov[,5])))

        }else{#outcome model 4: Y ~ A + M + C + M * (one of C)
            p_y1m0[,scenario] <- 1/(1+exp(-(-0.71 +0.61*1 +1.63*m0_all[,scenario]
                                            -1.39*cov[,1] -1.08*cov[,2] -0.75*cov[,3]
                                            -1.09*cov[,4] +0.60*cov[,5] +0.35*cov[,6]
                                            -1.45*m0_all[,scenario]*cov[,5])))
        }
}

# y1m0_all contains the binary outcome value of potential Y1M0 in all scenarios
for (scenario in 1:8 ){
        y1m0_all[,scenario] <- rbinom(N, 1, p_y1m0[,scenario])
}

# Y0M1
p_y0m1 <- data.frame(matrix(NA, nrow = N, ncol = 4))
y0m1_all <- data.frame(matrix(NA, nrow = N, ncol = 4))
for (scenario in 1:8){
        if (scenario %in% c(1,5)){#outcome model 1: Y ~ A + M + C
            p_y0m1[,scenario] <- 1/(1+exp(-(-0.61 +0.62*0 +2.0*m1_all[,scenario]
                                            -1.42*cov[,1] -1.08*cov[,2] -0.77*cov[,3]
                                            -1.08*cov[,4] +0.51*cov[,5] +0.36*cov[,6])))

        }else if (scenario %in% c(2,6)){#outcome model 2: Y ~ A + M + C + A*M
            p_y0m1[,scenario] <- 1/(1+exp(-(-0.61 +0.65*0 +1.7*m1_all[,scenario]
                                            -1.42*cov[,1] -1.07*cov[,2] -0.77*cov[,3]
```

```
                                        -1.07*cov[,4] -0.28*cov[,5] +0.36*cov[,6]
                                        -1.0*0*m1_all[,scenario])))

        }else if (scenario %in% c(3,7)){#outcome model 3: Y ~ A + M + C + A* (one of C)
                p_y0m1[,scenario] <- 1/(1+exp(-(-0.61 +0.64*0 +1.8*m1_all[,scenario]
                                        -1.42*cov[,1] -1.08*cov[,2] -0.78*cov[,3]
                                        -1.08*cov[,4] +0.50*cov[,5] +0.36*cov[,6]
                                        -1.2*0*cov[,5])))

        }else{#outcome model 4: Y ~ A + M + C + M * (one of C)
                p_y0m1[,scenario] <- 1/(1+exp(-(-0.71 +0.61*0 +1.63*m1_all[,scenario]
                                        -1.39*cov[,1] -1.08*cov[,2] -0.75*cov[,3]
                                        -1.09*cov[,4] +0.60*cov[,5] +0.35*cov[,6]
                                        -1.45*m1_all[,scenario]*cov[,5])))
        }
}

# y0m1_all contains the binary outcome value of potential Y0M1 in all scenarios
for (scenario in 1:8 ){
        y0m1_all[,scenario] <- rbinom(N, 1, p_y0m1[,scenario])
}



### comparison of E(Y) between counterfactuals
TE <- rep(NA, 8)
NDE0 <- rep(NA, 8)
NIE1 <- rep(NA, 8)
NDE1 <- rep(NA, 8)
NIE0 <- rep(NA, 8)

#QUESTION: just the raw mean difference???
for (scenario in 1:8){
        TE[scenario] <- mean(y1m1_all[,scenario]) - mean(y0m0_all[,scenario])
        NDE0[scenario] <- mean(y1m0_all[,scenario]) - mean(y0m0_all[,scenario])
        NIE1[scenario] <- mean(y1m1_all[,scenario]) - mean(y1m0_all[,scenario])
        NDE1[scenario] <- mean(y1m1_all[,scenario]) - mean(y0m1_all[,scenario])
```

```
        NIE0[scenario] <- mean(y0m1_all[,scenario]) - mean(y0m0_all[,scenario])
}

# assemble the true effects in a data frame
eff_true <- data.frame(matrix(NA, nrow = 5, ncol = 8))
rownames(eff_true) <- c("TE", "NDE0", "NIE1", "NDE1","NIE0")
colnames(eff_true) <- c("Sce 1", "Sce 2", "Sce 3", "Sce 4", "Sce 5", "Sce 6", "Sce 7", "Sce 8")
eff_true[1,] <- TE
eff_true[2,] <- NDE0
eff_true[3,] <- NIE1
eff_true[4,] <- NDE1
eff_true[5,] <- NIE0
eff_true
# Connection with Compute seems very unstable. Export the results.
write.csv(eff_true, "/home/tuo70113/simulation_project/output_sim/eff_true.csv")

## get the "observed" Y and M among the potential outcomes
y_obs <- data.frame(matrix(NA, nrow = N, ncol = 8))
m_obs <- data.frame(matrix(NA, nrow = N, ncol = 8))
for (scenario in 1:8){
        m_obs[,scenario] <- ifelse(treat_all[,scenario] == 0, m0_all[,scenario], m1_all[,scenario])
        y_obs[,scenario] <- ifelse(treat_all[,scenario] == 0, y0m0_all[,scenario], y1m1_all[,scenario])
}

##### Script 3: re_sim_esti_Haoyu.R
#### Estimate TRUE effects (in the population)
library(twangMediation)
library(twang)
library(foreach)
library(doParallel)

###Est 2
# re-estimate the propensity scores and use these to calculate total effect weights
# still do this on the observed data among the potential outcomes.
for (scenario in 1:8){
        data_pop[[scenario]]$outcome_obs <- y_obs[,scenario]
```

```
        data_pop[[scenario]]$mediator_obs <- m_obs[,scenario]
}
head(data_pop[[1]])
rm(y_obs)
rm(m_obs)

# mean centering X2 to make the model estimates more stable(less influenced by seed)
for (scenario in 1:8){
        data_pop[[scenario]]$X2_ctr <- data_pop[[scenario]]$X2 - mean(data_pop[[scenario]]$X2)
}

# create a variable equal to mediator*X2
for (scenario in 5:8) {
        data_pop[[scenario]]$mediator_X2 <-
                data_pop[[scenario]]$mediator_obs * data_pop[[scenario]]$X2_ctr
}
head(data_pop[[5]])

# propensity score models
ps_model <- vector("list", length = length(data_pop))
# scenarios 1 to 4
start_time <- Sys.time()
numcores<- detectCores()
registerDoParallel(floor(numcores/2)-1)
ps_model[1:4]<- foreach(scenario = 1:4, .verbose = T) %dopar% {
        ps_tmp <- glm(formula = treat~U1 + U2 + U3 + X1 + X2 + X3,
                      data = data_pop[[scenario]], family = "binomial")
        return(ps_tmp)
}
stopImplicitCluster()
end_time <- Sys.time()
end_time - start_time
#scenarios 5 to 8
# add the interaction term of M*X2 as a new column
#for (scenario in 5:8){
#       data_pop[[scenario]]$m_obs_X2 <- data_pop[[scenario]]$mediator_obs * data_pop[[scenario]]$X2
```

```
#}
#table(data_pop[[5]]$m_obs_X2)

start_time <- Sys.time()
numcores<- detectCores()
registerDoParallel(floor(numcores/2)-1)
ps_model[5:8]<-  foreach(scenario = 5:8, .verbose = T) %dopar% {
        ps_tmp <- glm(formula = treat~U1 + U2 + U3 + X1 + X2 + X3,
                      data = data_pop[[scenario]], family = "binomial")
        return(ps_tmp)
}
stopImplicitCluster()
end_time <- Sys.time()
end_time - start_time



# predict propensity scores
ps_all <- data.frame(matrix(data = NA, nrow = N, ncol = 8))
for (scenario in 1:8){
        ps_all[,scenario] <- predict(ps_model[[scenario]],
                                data_pop[[scenario]][, c("U1", "U2", "U3", "X1", "X2", "X3")],
                                type = "response")
}
#check true PS and estimated PS
plot(y = p_treat, x = ps_all[,5])

# calculate the NEW total effect weights using estimated propensity scores
for (scenario in 1:8){
        data_pop[[scenario]]$weight_te_obs <-
                ifelse(treat_all[,scenario] ==1, 1/ps_all[,scenario], 1/(1-ps_all[,scenario]))

}
# manually check TE in scenario 1
data5 <- data_pop[[5]]
te5 <- mean(data5[data5$treat == 1,]$outcome_obs * data5[data5$treat == 1,]$weight_te_obs)/
```

```
mean(data5[data5$treat == 1,]$weight_te_obs) -
        mean(data5[data5$treat == 0,]$outcome_obs * data5[data5$treat == 0,]$weight_te_obs)/
        mean(data5[data5$treat == 0,]$weight_te_obs)
te5
rm(data5)
rm(te5)

## true mediated effect estimates in the observed data among potential outcomes
medtrue_obs2 <- vector("list", length = length(data_pop))
# run estimation in parallel
start_time <- Sys.time()
numcores<- detectCores()
registerDoParallel(floor(numcores/2)-1)
medtrue_obs2[1:4]<- foreach(scenario = 1:4, .verbose = T) %dopar% {
        eff <- wgtmed(formula.med = mediator_obs~U1+U2+U3+X1+X2+X3,
                      a_treatment = "treat", y_outcome = "outcome_obs",
                      data = data_pop[[scenario]], method = "logistic",
                      total_effect_wts = data_pop[[scenario]]$weight_te_obs)
        return(eff)
}
stopImplicitCluster()
end_time <- Sys.time()
end_time - start_time

start_time <- Sys.time()
registerDoParallel(numcores-4)
medtrue_obs2[5:8]<- foreach(scenario = 5:8, .verbose = T) %dopar% {
        eff <- wgtmed(formula.med = mediator_obs~U1+U2+U3+X1+X2_ctr+X3+mediator_X2,
                      a_treatment = "treat", y_outcome = "outcome_obs",
                      med_interact = "mediator_X2",
                      data = data_pop[[scenario]], method = "logistic",
                      total_effect_wts = data_pop[[scenario]]$weight_te_obs)
        return(eff)
}
stopImplicitCluster()
end_time <- Sys.time()
```

```
end_time - start_time

## print true effect estimates of the observed among the potential outcomes
for(scenario in 1:length(data_pop)){
        cat("Scenario:", scenario, "\n")
        summary(medtrue_obs2[[scenario]])
        cat("\n")
}

# store the weights and population_based effect estimates
# for analysis in sim_hpc_part2.R
y_pop <- data.frame(matrix(NA, nrow = 4, ncol = 8))
effects_pop <- data.frame(matrix(NA, nrow = 5, ncol = 8))
for (sce in 1:8){
        w11 <- attr(medtrue_obs2[[sce]], "w_11")
        w00 <- attr(medtrue_obs2[[sce]], "w_00")
        w10 <- attr(medtrue_obs2[[sce]], "w_10")
        w01 <- attr(medtrue_obs2[[sce]], "w_01")
        pop_tmp <- data_pop[[sce]]
        #pop y00 estimates
        y_pop[1,sce] <- mean(pop_tmp[pop_tmp$treat ==0, ]$outcome_obs * w00[!is.na(w00)]/
        mean(w00, na.rm = TRUE))
        #pop y11 estimates
        y_pop[2,sce] <- mean(pop_tmp[pop_tmp$treat ==1, ]$outcome_obs * w11[!is.na(w11)]/
        mean(w11, na.rm = TRUE))
        #pop y10 estimates
        y_pop[3,sce] <- mean(pop_tmp[pop_tmp$treat ==1, ]$outcome_obs * w10[!is.na(w10)]/
        mean(w10, na.rm = TRUE))
        #pop y01 estimates
        y_pop[4,sce] <- mean(pop_tmp[pop_tmp$treat ==0, ]$outcome_obs * w01[!is.na(w01)]/
        mean(w01, na.rm = TRUE))

        # effects
        effects_pop[1, sce] <- desc.effects(medtrue_obs2[[sce]])[[1]][1,1]
        effects_pop[2, sce] <- desc.effects(medtrue_obs2[[sce]])[[1]][2,1]
        effects_pop[3, sce] <- desc.effects(medtrue_obs2[[sce]])[[1]][3,1]
```

```
        effects_pop[4, sce] <- desc.effects(medtrue_obs2[[sce]])[[1]][4,1]
        effects_pop[5, sce] <- desc.effects(medtrue_obs2[[sce]])[[1]][5,1]
}
rownames(y_pop) <- c("Y_00", "Y_11", "Y_10", "Y_01")
colnames(y_pop) <- 1:8
rownames(effects_pop) <- c("TE", "NDE_0", "NIE_1", "NDE_1", "NIE_0")
colnames(effects_pop) <- 1:8

##### Script 4: samplling_sim.R
## Sampling of the simulation project
#02/08/2022
# Haoyu (Tophey) Zhou
library(doParallel)
library(dplyr)
set.seed(100)
#### test sample selection models
## sampling scenario 1 S ~ U
for (scenario in 1:8) {
        data_pop[[scenario]]$p_sel1 <- 1/(1+exp(-(0.3 - 1.39*U1 - 1.28*U2 +1.13 *U3)))
        data_pop[[scenario]]$smp_wgt1 <- (1/data_pop[[scenario]]$p_sel1)
}

## sampling scenario 2 S ~ U + M
for (scenario in 1:8) {
        data_pop[[scenario]]$p_sel2 <-
                1/(1+exp(-(0.3 - 1.39*U1 - 1.39*U2 +1.23*U3 + 1.45*data_pop[[scenario]]$mediator_obs)))
        data_pop[[scenario]]$smp_wgt2 <- (1/data_pop[[scenario]]$p_sel2)
}
## sampling scenario 3 S ~ U + A
for (scenario in 1:8) {
        data_pop[[scenario]]$p_sel3 <-
                1/(1+exp(-(0.3 - 1.39*U1 - 1.23*U2 + 1.3*U3 + 1.45*treat_all[[scenario]])))
        data_pop[[scenario]]$smp_wgt3 <- (1/data_pop[[scenario]]$p_sel3)
}
## sampling scenario 4 simple random sampling
for (scenario in 1:8){
```

```
        data_pop[[scenario]]$p_sel4 <- 9000/N
}


head(data_pop[[1]])

## test 3 sampling scenarios for each sampling scenario
## draw 3 repeated samples

#Steps: compute the ps and sampling weights, then get the complete sample data
# sampling weights of repeated samples
n_iter <- 1000
idsel <- vector("list", n_iter) #id of the selected
sw <- vector("list", 3)
psel <- vector('list', 3) # asmpling probability of the selected
for (i in 1:n_iter)( # i = ith repeated sampling
        idsel[[i]] <- data.frame(matrix(NA, nrow = 9000, ncol = 24))
)
for (i in 1:n_iter){
        sw[[i]] <- data.frame(matrix(NA, nrow = 9000, ncol = 24))
}
for (i in 1:n_iter){
        psel[[i]] <- data.frame(matrix(NA, nrow = 9000, ncol = 24))
}

# get the sampled IDs
set.seed(200)

# run the generation of idsel in parallel
idsel_tmp <- data.frame(matrix(NA, nrow = 9000, ncol = 32))
start_time <- Sys.time()
numcores<- detectCores()
registerDoParallel(floor(numcores/2)-1)
idsel <- foreach(i = 1:n_iter, .verbose = T) %dopar% {
        for (s_sce in 1:4){ # 4 sampling scenarios
                for (scenario in 1:8){ # 3 population scenarios
```

```
                        if (s_sce ==1){
                                idsel_tmp[,(s_sce-1)*8+scenario] <-
                                        sample(N, 9000, prob =
                                                       data_pop[[scenario]]$p_sel1, replace = F)
                        }else if (s_sce == 2){idsel_tmp[,(s_sce-1)*8+scenario] <-
                                sample(N, 9000, prob =
                                               data_pop[[scenario]]$p_sel2, replace = F)
                        }else if (s_sce == 3){idsel_tmp[,(s_sce-1)*8+scenario] <-
                                sample(N, 9000, prob =
                                               data_pop[[scenario]]$p_sel3, replace = F)
                        }else(idsel_tmp[,(s_sce-1)*8+scenario] <-
                                      sample(N, 9000, prob = data_pop[[scenario]]$p_sel4, replace = F))
                }
        }
        return(idsel_tmp)
}
end_time <- Sys.time()
end_time - start_time

# sampling probability and weights of the selected
for(i in 1:n_iter){ # 3 repeated samples
        for (s_sce in 1:4){ # 3 sampling scenarios
                for (scenario in 1:8){ # 3 population scenarios
                        if (s_sce ==1){
                                p_seltmp <- data_pop[[scenario]]$p_sel1
                                psel[[i]][, (s_sce-1)*8+scenario] <-
                                        p_seltmp[idsel[[i]][,(s_sce-1)*8+scenario]]
                        }else if (s_sce == 2){
                                p_seltmp <- data_pop[[scenario]]$p_sel2
                                psel[[i]][, (s_sce-1)*8+scenario] <-
                                        p_seltmp[idsel[[i]][,(s_sce-1)*8+scenario]]
                        }else if (s_sce ==3){p_seltmp <- data_pop[[scenario]]$p_sel3
                        psel[[i]][, (s_sce-1)*8+scenario] <- p_seltmp[idsel[[i]][,(s_sce-1)*8+scenario]]
                        }else{p_seltmp <- data_pop[[scenario]]$p_sel4
                        psel[[i]][, (s_sce-1)*8+scenario] <-
                                p_seltmp[idsel[[i]][,(s_sce-1)*8+scenario]]
```

```
                        }
                }
        }
}

# now psel contains all the sampling probabilities of the individuals in samples
# calculate sampling weights
for (i in 1:n_iter){
        sw[[i]] <- 1/psel[[i]]
}

# sampling weights can be generated
# before the computationally intensive iterations
# Reassemble the sampling weights so that each condition is saved in one data frame
# add a simple random sampling to sw in the mean time
# do the same reassembling with idsel
sw_tmp <- vector("list", 32)
id_tmp <- vector("list", 32)
for (s_sce in 1:32){# 4 sampling * 8 population data scenarios
        sw_tmp[[s_sce]] <- data.frame(matrix(NA, nrow = 9000, ncol = n_iter))
        id_tmp[[s_sce]] <- data.frame(matrix(NA, nrow = 9000, ncol = n_iter))
        for (i in 1:n_iter){
                sw_tmp[[s_sce]][, i] <- sw[[i]][,s_sce]
                id_tmp[[s_sce]][, i] <- idsel[[i]][,s_sce]
        }
}
sw_re<- vector("list", 32)
id_re <- vector("list", 32)
for (i in 1:32){
        sw_re[[(i - (i-1)%/%8 * 8 -1)*4+ 1+(i-1)%/%8]] <- sw_tmp[[i]]
        id_re[[(i - (i-1)%/%8 * 8 -1)*4+ 1+(i-1)%/%8]] <- id_tmp[[i]]
}

# concatenate sw_re and id_re into single data frames and export them
id_retable <- data.frame(matrix(NA, nrow = 32*9000, ncol = n_iter))
```

```
sw_retable <- data.frame(matrix(NA, nrow = 32*9000, ncol = n_iter))
for (i in 1:32){
        id_tmp <- id_re[[i]]
        sw_tmp <- sw_re[[i]]
        if (i ==1 ){
                id_retable <- id_tmp
                sw_retable <- sw_tmp
        }else{
                id_retable <- rbind(id_retable, id_tmp)
                sw_retable <- rbind(sw_retable, sw_tmp)
        }
}
write.csv(id_retable, file = "./id_retable.csv")
write.csv(sw_retable, file = "./sw_retable.csv")
write_csv(x = data_pop[[1]], file = "./scenario1.csv")
write_csv(x = data_pop[[2]], file = "./scenario2.csv")
write_csv(x = data_pop[[3]], file = "./scenario3.csv")
write_csv(x = data_pop[[4]], file = "./scenario4.csv")
write_csv(x = data_pop[[5]], file = "./scenario5.csv")
write_csv(x = data_pop[[6]], file = "./scenario6.csv")
write_csv(x = data_pop[[7]], file = "./scenario7.csv")
write_csv(x = data_pop[[8]], file = "./scenario8.csv")
write_csv(y1m1_all, file = "./y1m1_all.csv")
write_csv(y0m0_all, file = "./y0m0_all.csv")
write_csv(y1m0_all, file = "./y1m0_all.csv")
write_csv(y0m1_all, file = "./y0m1_all.csv")
write_csv(y_pop, file = "./y_pop.csv")
write_csv(effects_pop, file ="./effects_pop.csv")

end_time1 <- Sys.time()
end_time1 - start_time1




start_time2 <- Sys.time()
##### Script 5: Iteration2.R
```

```
### put all the sample statistics in a data frame
library(doParallel)
library(twang)
library(twangMediation)
library(writexl)
library(dplyr)
library(readr)

#sample size
N <- 90000
# # of iterations
n_iter <- 1000

# import results exported in sim_hpc_part1.R
# because we break down the scripts into part 1 and part 2
# and Rscript does not store results in the memory
eff_true<- read.csv("./eff_true.csv")
id_retable <- read.csv("./id_retable.csv")
sw_retable <- read.csv("./sw_retable.csv")
data_pop <- vector("list", 8)
data_pop[[1]] <- read.csv("./scenario1.csv")
data_pop[[2]] <- read.csv("./scenario2.csv")
data_pop[[3]] <- read.csv("./scenario3.csv")
data_pop[[4]] <- read.csv("./scenario4.csv")
data_pop[[5]] <- read.csv("./scenario5.csv")
data_pop[[6]] <- read.csv("./scenario6.csv")
data_pop[[7]] <- read.csv("./scenario7.csv")
data_pop[[8]] <- read.csv("./scenario8.csv")
y1m1_all <- read.csv("./y1m1_all.csv")
y0m0_all <- read.csv("./y0m0_all.csv")
y1m0_all <- read.csv("./y1m0_all.csv")
y0m1_all <- read.csv("./y0m1_all.csv")
y_pop <- read.csv("./y_pop.csv")
effects_pop <-read.csv("./effects_pop.csv")

row.names(eff_true) <- eff_true[,1]
```

```
eff_true <- eff_true[,2:ncol(eff_true)]
row.names(id_retable) <- id_retable[,1]
id_retable <- id_retable[,2:ncol(id_retable)]
row.names(sw_retable) <- sw_retable[,1]
sw_retable <- sw_retable[,2:ncol(sw_retable)]


# re-organize id_retable and sw_retable into lists,
# for the way I coded.
id_re <- vector("list", 32)
sw_re <- vector("list", 32)
for (i in 1:32){
        id_re[[i]] <- id_retable[((i-1)*9000+1):(9000*i),]
        sw_re[[i]] <- sw_retable[((i-1)*9000+1):(9000*i),]
}

# a data frame to store the summary results
sum_table <- data.frame(matrix(data = NA, nrow = 16*8, ncol = 94))
colnames(sum_table) <- c("scenario", "sampling", "weight_strategy","mean_swde",
                         "mean_te", "bias_te", "rbias_te", "bias2_te","mse_te", "sd_te", "meanse_te",
                         "mean_nde0", "bias_nde0", "rbias_nde0", "bias2_nde0",
                         "mse_nde0", "sd_nde0", "meanse_nde0",
                         "mean_nie1", "bias_nie1", "rbias_nie1", "bias2_nie1",
                         "mse_nie1", "sd_nie1", "meanse_nie1",
                         "mean_nde1", "bias_nde1", "rbias_nde1", "bias2_nde1",
                         "mse_nde1", "sd_nde1", "meanse_nde1",
                         "mean_nie0", "bias_nie0", "rbias_nie0", "bias2_nie0",
                         "mse_nie0", "sd_nie0", "meanse_nie0",
                         "min_ess00","mean_ess00", "max_ess00", "min_ess11",
                         "mean_ess11", "max_ess11",
                         "min_ess10", "mean_ess10", "max_ess10",
                         "min_ess01","mean_ess01", "max_ess01",
                         "min_ess00_b","mean_ess00_b", "max_ess00_b",
                         "min_ess11_b","mean_ess11_b", "max_ess11_b",
                         "min_ess10_b","mean_ess10_b", "max_ess10_b",
                         "min_ess01_b","mean_ess01_b", "max_ess01_b",
```

```
                          "meanchi_treatmed", "meanchi_treatout", "meanchi_medout",
                          "mean_y00", "bias_y00", "rbias_y00", "bias2_y00", "mse_y00",
                          "sd_y00", "meanse_y00",
                          "mean_y11", "bias_y11", "rbias_y11", "bias2_y11", "mse_y11",
                          "sd_y11", "meanse_y11",
                          "mean_y10", "bias_y10", "rbias_y10", "bias2_y10", "mse_y10",
                          "sd_y10", "meanse_y10",
                          "mean_y01", "bias_y01", "rbias_y01", "bias2_y01", "mse_y01",
                          "sd_y01", "meanse_y01")

sum_table$scenario <- c(rep(1, 16), rep(2, 16), rep(3,16), rep(4,16),
                        rep(5,16), rep(6, 16), rep(7, 16), rep(8, 16))
sum_table$sampling <- rep(c(rep(1, 4), rep(2, 4), rep(3,4), rep(4,4)), 8)
sum_table$weight_strategy <- rep(1:4, 32)

## ALSO save the statistics of each iteration
# Eventually 128 tables like iter_stats in iter_stats_list
# each table contains the statistics of n_iter iterations
# under one combination of scenarios, samplings, and weightings
iter_stats <- data.frame(matrix(NA, nrow = n_iter, ncol = 33))
colnames(iter_stats) <- c("scenario", "sampling","weight_strategy", "swde","te",
"se_te", "nde0", "se_nde0",
                          "nie1", "se_nie1","nde1", "se_nde1",
                          "nie0", "se_nie0", "y00", "se_y00",
                          "y11", "se_y11","y10", "se_y10",
                          "y01", "se_y01", "chi_treatmed", "chi_treatout", "chi_medout",
                          "ess00", "ess11", "ess10", "ess01",
                          "ess00_b", "ess11_b", "ess10_b", "ess01_b")

# weighting strategy: (1/0) use sampling weight in estimating propensity score
# (1/0) use sampling weight in estimating the treatment effects
# 1: 00.        2:01.           3:10.    4:11

# sample size
n <- 9000
numcores <- detectCores()
```

```
## let foreach() output a list, each row of which is a data frame with # of rows == n_iter
iter_stats_list <- vector("list", 128)
start_time <- Sys.time()
registerDoParallel(floor(numcores/2)-1)
iter_stats_list <- foreach(r = 1:128, .verbose = T) %dopar% { #128 rows in sample_stats
        sce <- (r-1)%/%16 + 1 # identify the population scenario
        smp <- (r-1)%%16 %/% 4 +1 # identify the sampling scenario
        wstrat <- (r-1)%%4 +1 # identify the weighting strategy

        for (i in 1:n_iter){ # loop over n_iter iterations
                #sample_tmp <- data_pop[[sce]][id_re[[(r-1)*4+1]][,i],]
                #(r-1)*4+1 is the corresponding indicator in id_re
                sample_tmp <- data_pop[[sce]][id_re[[(sce-1)*4+smp]][,i],] #test run
                # remove unnecessary columns from the sample
                sample_tmp <- sample_tmp %>%
                        select(treat, U1, U2, U3, X1, X2, X2_ctr, X3, outcome_obs, mediator_obs)
                if (sce %in% 5:8){
                        sample_tmp$mediator_X2 <- sample_tmp$mediator_obs * sample_tmp$X2_ctr
                }
                # (still within the for loop)
                sample_tmp$sw <- sw_re[[(sce-1)*4+smp]][,i]
                if (wstrat == 1 | wstrat == 2){
                        # estimate ps
                        ps_model_tmp <- glm(formula = treat~U1 + U2 + U3 + X1 + X2 + X3,
                                            data = sample_tmp, family = "binomial")
                }else{  ps_model_tmp <- glm(formula = treat~U1 + U2 + U3 + X1 + X2 + X3,
                                            data = sample_tmp, family = "binomial",
                                            weights = sample_tmp$sw)
                }
                sample_tmp$ps <- predict(ps_model_tmp,
                                    sample_tmp[,c("U1", "U2", "U3", "X1", "X2", "X3")],
                                    type = "response")
                # inverse probability weights
                sample_tmp$tew <- ifelse(sample_tmp$treat ==1, 1/sample_tmp$ps, 1/(1-sample_tmp$ps))
                # estimate mediated effects
```

```
if(sce %in% 1:4){
        med_tmp <- wgtmed(formula.med = mediator_obs~U1+U2+U3+X1+X2+X3,
                          a_treatment = "treat", y_outcome = "outcome_obs",
                          data = sample_tmp, method = "logistic",
                          total_effect_wts = sample_tmp$tew,
                          sampw = ifelse(rep(wstrat == 1| wstrat == 2, n),
                          rep(1,n), sample_tmp$sw))
        # ifelse returns the element of the same length as the "test" argument
}else{# interaction term for sce 5:8
        med_tmp <-wgtmed(formula.med = mediator_obs~U1+U2+U3+X1+X2_ctr+X3+mediator_X2,
                         a_treatment = "treat", y_outcome = "outcome_obs",
                         med_interact = "mediator_X2",
                         data = sample_tmp, method = "logistic",
                         total_effect_wts = sample_tmp$tew,
                         sampw = ifelse(rep(wstrat == 1| wstrat == 2, n),
                         rep(1,n), sample_tmp$sw))
}
# save the statistics of one iteration
if (wstrat == 2){ # 2:01
        attr(med_tmp, "sampw") <- sample_tmp$sw
        }
else if(wstrat == 3) { # 3:10
        attr(med_tmp, "sampw") <- rep(1,n)
}
sum_tmp <- desc.effects(med_tmp)[[1]]
est_tmp <- round(sum_tmp, 4)[,c(1,2)]# keep only effect and std.err
te <- est_tmp[1,1]
se_te <- est_tmp[1,2]
nde0 <- est_tmp[2,1]
se_nde0 <- est_tmp[2,2]
nie1 <- est_tmp[3,1]
se_nie1 <- est_tmp[3,2]
nde1 <- est_tmp[4,1]
se_nde1 <- est_tmp[4,2]
nie0 <- est_tmp[5,1]
se_nie0 <- est_tmp[5,2]
```

```
#design effect of sampling weight
swde <- 1 + (sd(sample_tmp$sw)/mean(sample_tmp$sw))^2

# potential outcomes
# first add w11, w00, w10, w01 to sample_tmp
# obtain estimated weights
w00 <- ifelse(rep(wstrat ==2 | wstrat == 4, n), attr(med_tmp, "w_00")* sample_tmp$sw,
              attr(med_tmp, "w_00"))
w11 <- ifelse(rep(wstrat ==2 | wstrat == 4, n), attr(med_tmp, "w_11")* sample_tmp$sw,
              attr(med_tmp, "w_11"))
w10 <- ifelse(rep(wstrat ==2 | wstrat == 4, n), attr(med_tmp, "w_10")* sample_tmp$sw,
              attr(med_tmp, "w_10"))
w01 <- ifelse(rep(wstrat ==2 | wstrat == 4, n), attr(med_tmp, "w_01")* sample_tmp$sw,
              attr(med_tmp, "w_01"))

# with update to twangMediation, the four w's are the products of cross-world weights
# and sampling weights when sampw is used in wgtmed()
# calculate  potential outcomes using the four w's
indi_y11<- sample_tmp[sample_tmp$treat ==1,]$outcome_obs *
        w11[!is.na(w11)]/mean(w11, na.rm = TRUE)
indi_y00<- sample_tmp[sample_tmp$treat ==0,]$outcome_obs *
        w00[!is.na(w00)]/mean(w00, na.rm = TRUE)
indi_y10<- sample_tmp[sample_tmp$treat ==1,]$outcome_obs *
        w10[!is.na(w10)]/mean(w10, na.rm = TRUE)
indi_y01<- sample_tmp[sample_tmp$treat ==0,]$outcome_obs *
        w01[!is.na(w01)]/mean(w01, na.rm = TRUE)

y11 <- mean(indi_y11)
y00 <- mean(indi_y00)
y10 <- mean(indi_y10)
y01 <- mean(indi_y01)
## caculate standard error of potential ys
indi_y11obs <- sample_tmp[sample_tmp$treat ==1,]$outcome_obs
indi_y00obs <- sample_tmp[sample_tmp$treat ==0,]$outcome_obs
r11 <- indi_y11obs - sum(w11[!is.na(w11)]*indi_y11obs)/sum(w11[!is.na(w11)])
```

```
r00 <- indi_y00obs - sum(w00[!is.na(w00)]*indi_y00obs)/sum(w00[!is.na(w00)])
r10 <- indi_y11obs - sum(w10[!is.na(w10)]*indi_y11obs)/sum(w10[!is.na(w10)])
r01 <- indi_y00obs - sum(w01[!is.na(w01)]*indi_y00obs)/sum(w01[!is.na(w01)])
# actually the term after - is just y11, y00, y10, and y01 I calculated above

# SE of potential ys
se_y11 <- sqrt(n/(n-1) *sum(w11[!is.na(w11)]^2 * r11^2)/ (sum(w11[!is.na(w11)]))^2)
se_y00 <- sqrt(n/(n-1) *sum(w00[!is.na(w00)]^2 * r00^2)/ (sum(w00[!is.na(w00)]))^2)
se_y10 <- sqrt(n/(n-1) *sum(w10[!is.na(w10)]^2 * r10^2)/ (sum(w10[!is.na(w10)]))^2)
se_y01 <- sqrt(n/(n-1) *sum(w01[!is.na(w01)]^2 * r01^2)/ (sum(w01[!is.na(w01)]))^2)

# 3 chi-square statistics
chi_treatmed <- chisq.test(sample_tmp$treat, sample_tmp$mediator_obs)$statistic
chi_treatout <- chisq.test(sample_tmp$treat, sample_tmp$outcome_obs)$statistic
chi_medout <- chisq.test(sample_tmp$mediator_obs, sample_tmp$outcome_obs)$statistic

# ESS of cross-world weights and of total effect weights
ess <- summary(med_tmp)$ess_table
ess00 <- ess[1,1]
ess11 <- ess[1,2]
ess10 <- ess[1,3]
ess01 <- ess[1,4]
# ESS that incorporates both mediation weights and sampling weights
# for weighting strategy 2 and 4 only.
if (wstrat == 2 | wstrat == 4){
        ess00_b <- sum(w00[!is.na(w00)])^2 / sum(w00[!is.na(w00)]^2)
        ess11_b <- sum(w11[!is.na(w11)])^2 / sum(w11[!is.na(w11)]^2)
        ess10_b <- sum(w10[!is.na(w10)])^2 / sum(w10[!is.na(w10)]^2)
        ess01_b <- sum(w01[!is.na(w01)])^2 / sum(w01[!is.na(w01)]^2)
}else{
        ess00_b <- NA
        ess11_b <- NA
        ess10_b <- NA
        ess01_b <- NA
}
```

```
            # Now assemble the statistics in the correlated row in the data frame
            # of this combination of scenarios, sampling, and weighting strategies
            iter_stats[i,] <- c(sce, smp, wstrat,swde, te, se_te, nde0, se_nde0,
                                    nie1, se_nie1, nde1, se_nde1,
                                    nie0, se_nie0, y00, se_y00,
                                    y11, se_y11, y10, se_y10,
                                    y01, se_y01, chi_treatmed, chi_treatout, chi_medout,
                                    ess00, ess11, ess10, ess01, ess00_b, ess11_b, ess10_b, ess01_b)
        }### end of the for loop
        return(iter_stats)
}
stopImplicitCluster()
end_time <- Sys.time()
end_time - start_time #59.86328 mins for 10 iterations

# calculate bias (absolute and relative)
iter_stats_table <- data.frame(matrix(NA, nrow = 128000))
for (r in 1:128) {
        sce <- (r-1)%/%16 + 1 # identify the population scenario
        iter_stats <- iter_stats_list[[r]]
        iter_stats$bias_te <- iter_stats$te - eff_true[1,sce]
        iter_stats$bias_nde0 <- iter_stats$nde0 - eff_true[2,sce]
        iter_stats$bias_nie1 <- iter_stats$nie1 - eff_true[3,sce]
        iter_stats$bias_nde1 <- iter_stats$nde1 - eff_true[4,sce]
        iter_stats$bias_nie0 <- iter_stats$nie0 - eff_true[5,sce]
        iter_stats$bias_y00 <- iter_stats$y00 - mean(y0m0_all[,sce])
        iter_stats$bias_y11 <- iter_stats$y11 - mean(y1m1_all[,sce])
        iter_stats$bias_y10 <- iter_stats$y10 - mean(y1m0_all[,sce])
        iter_stats$bias_y01 <- iter_stats$y01 - mean(y0m1_all[,sce])

        # add relative bias. 05/12/2022
        iter_stats$rbias_te <- iter_stats$bias_te / eff_true[1,sce]
        iter_stats$rbias_nde0 <- iter_stats$bias_nde0 / eff_true[2,sce]
        iter_stats$rbias_nie1 <- iter_stats$bias_nie1 / eff_true[3,sce]
        iter_stats$rbias_nde1 <- iter_stats$bias_nde1 / eff_true[4,sce]
        iter_stats$rbias_nie0 <- iter_stats$bias_nie0 / eff_true[5,sce]
```

```
        iter_stats$rbias_y00 <- iter_stats$bias_y00 / mean(y0m0_all[,sce])
        iter_stats$rbias_y11 <- iter_stats$bias_y11 / mean(y1m1_all[,sce])
        iter_stats$rbias_y01 <- iter_stats$bias_y01 / mean(y0m1_all[,sce])
        iter_stats$rbias_y10 <- iter_stats$bias_y10 / mean(y1m0_all[,sce])
        iter_stats_list[[r]] <- iter_stats
}

# rbind the statistics of 128 data frames
# add new bias2: sample_based estimates - population-based estimates. 05/15/2022
for (r in 1:128){
        sce <- (r-1)%/%16 + 1 # identify the population scenario
        iter_stats <- iter_stats_list[[r]]
        iter_stats$bias2_te <- iter_stats$te - effects_pop[1,sce]
        iter_stats$bias2_nde0 <- iter_stats$nde0 - effects_pop[2,sce]
        iter_stats$bias2_nie1 <- iter_stats$nie1 - effects_pop[3,sce]
        iter_stats$bias2_nde1 <- iter_stats$nde1 - effects_pop[4,sce]
        iter_stats$bias2_nie0 <- iter_stats$nie0 - effects_pop[5,sce]
        iter_stats$bias2_y00 <- iter_stats$y00 - y_pop[1, sce]
        iter_stats$bias2_y11 <- iter_stats$y11 - y_pop[2, sce]
        iter_stats$bias2_y10 <- iter_stats$y10 - y_pop[3, sce]
        iter_stats$bias2_y01 <- iter_stats$y01 - y_pop[4, sce]
        if (r == 1){
                iter_stats_table <- iter_stats
        }else(
                iter_stats_table <- rbind(iter_stats_table, iter_stats)
        )

}
iter_stats_table$sampling <- factor(iter_stats_table$sampling)
iter_stats_table$weight_strategy <- factor(iter_stats_table$weight_strategy)

write_csv(iter_stats_table, "/home/tuo70113/simulation_project/output_sim/iter_stats_table.csv")


#### Summarize each data frame (i.e. each combination of conditions) in iter_stats_list
# By default, foreach outputs a list
```

```
# Combine the elements of the list in a data frame afterwards
sum_stats_list <- vector("list", 128)
start_time <- Sys.time()
registerDoParallel(floor(numcores/2)-1)
sum_stats_list <- foreach(r = 1:128, .verbose = T) %dopar% {
        sce <- (r-1)%/%16 + 1 # identify the population scenario
        smp <- (r-1)%%16 %/% 4 +1 # identify the sampling scenario
        wstrat <- (r-1)%%4 +1 # identify the weighting strategy

        iter_stats<- iter_stats_list[[r]]
        # mean design effect of sampling weight
        mean_swde <- mean(iter_stats$swde)
        # te summary statistics
        mean_te <- mean(iter_stats$te)
        bias_te <- mean(iter_stats$te - eff_true[1,sce])
        rbias_te <- bias_te / eff_true[1,sce]
        bias2_te <- mean(iter_stats$te - effects_pop[1,sce])
        mse_te <- mean((iter_stats$te - eff_true[1,sce])^2)
        sd_te <- sd(iter_stats$te)
        meanse_te <- mean(iter_stats$se_te)
        # nde0 summary
        mean_nde0 <- mean(iter_stats$nde0)
        bias_nde0 <- mean(iter_stats$nde0 - eff_true[2, sce])
        rbias_nde0 <- bias_nde0 / eff_true[2, sce]
        bias2_nde0 <- mean(iter_stats$nde0 - effects_pop[2,sce])
        mse_nde0 <- mean((iter_stats$nde0 - eff_true[1,sce])^2 )
        sd_nde0 <- sd(iter_stats$nde0)
        meanse_nde0 <- mean(iter_stats$se_nde0)
        # nie1 summary
        mean_nie1 <- mean(iter_stats$nie1)
        bias_nie1 <- mean(iter_stats$nie1 - eff_true[3,sce])
        rbias_nie1 <- bias_nie1 / eff_true[3,sce]
        bias2_nie1 <- mean(iter_stats$nie1 - effects_pop[3,sce])
        mse_nie1 <- mean((iter_stats$nie1 - eff_true[3,sce])^2 )
        sd_nie1 <- sd(iter_stats$nie1)
        meanse_nie1 <- mean(iter_stats$se_nie1)
```

```
# nde1 summary
mean_nde1 <- mean(iter_stats$nde1)
bias_nde1 <- mean(iter_stats$nde1 - eff_true[4,sce])
rbias_nde1 <- bias_nde1 / eff_true[4,sce]
bias2_nde1 <- mean(iter_stats$nde1 - effects_pop[4,sce])
mse_nde1 <- mean((iter_stats$nde1 - eff_true[4,sce])^2 )
sd_nde1 <- sd(iter_stats$nde1)
meanse_nde1 <- mean(iter_stats$se_nde1)
# nie0 summary
mean_nie0 <- mean(iter_stats$nie0 )
bias_nie0  <- mean(iter_stats$nie0  - eff_true[5,sce])
rbias_nie0 <- bias_nie0/ eff_true[5,sce]
bias2_nie0 <- mean(iter_stats$nie0 - effects_pop[5,sce])
mse_nie0  <- mean((iter_stats$nie0  - eff_true[5,sce])^2 )
sd_nie0  <- sd(iter_stats$nie0 )
meanse_nie0  <- mean(iter_stats$se_nie0)
# ess00 summary
min_ess00 <- min(iter_stats$ess00)
mean_ess00 <- mean(iter_stats$ess00)
max_ess00 <- max(iter_stats$ess00)
# ess11 summary
min_ess11 <- min(iter_stats$ess11)
mean_ess11 <- mean(iter_stats$ess11)
max_ess11 <- max(iter_stats$ess11)
# ess10 summary
min_ess10 <- min(iter_stats$ess10)
mean_ess10 <- mean(iter_stats$ess10)
max_ess10 <- max(iter_stats$ess10)
# ess01 summary
min_ess01 <- min(iter_stats$ess01)
mean_ess01 <- mean(iter_stats$ess01)
max_ess01 <- max(iter_stats$ess01)

# ess00 summary
min_ess00_b <- min(iter_stats$ess00_b)
mean_ess00_b <- mean(iter_stats$ess00_b)
```

```
max_ess00_b <- max(iter_stats$ess00_b)
# ess11 summary
min_ess11_b <- min(iter_stats$ess11_b)
mean_ess11_b <- mean(iter_stats$ess11_b)
max_ess11_b <- max(iter_stats$ess11_b)
# ess10 summary
min_ess10_b <- min(iter_stats$ess10_b)
mean_ess10_b <- mean(iter_stats$ess10_b)
max_ess10_b <- max(iter_stats$ess10_b)
# ess01 summary
min_ess01_b <- min(iter_stats$ess01_b)
mean_ess01_b <- mean(iter_stats$ess01_b)
max_ess01_b <- max(iter_stats$ess01_b)

# mean of chi-square statistics
meanchi_treatmed <- mean(iter_stats$chi_treatmed)
meanchi_treatout <- mean(iter_stats$chi_treatout)
meanchi_medout <- mean(iter_stats$chi_medout)
# y00 summary
mean_y00 <- mean(iter_stats$y00)
bias_y00 <- mean_y00 - mean(y0m0_all[,sce])
rbias_y00 <- bias_y00 / mean(y0m0_all[,sce])
bias2_y00 <- mean_y00 - y_pop[1, sce]
mse_y00 <- mean((iter_stats$y00-y0m0_all[,sce])^2)
sd_y00 <- sd(iter_stats$y00)
meanse_y00 <- mean(iter_stats$se_y00)
# y11 summary
mean_y11 <- mean(iter_stats$y11)
bias_y11 <- mean_y11 - mean(y1m1_all[,sce])
rbias_y11 <- bias_y11/ mean(y1m1_all[,sce])
bias2_y11 <- mean_y11 - y_pop[2, sce]
mse_y11 <- mean((iter_stats$y11-y1m1_all[,sce])^2)
sd_y11 <- sd(iter_stats$y11)
meanse_y11 <- mean(iter_stats$se_y11)
# y10 summary
mean_y10 <- mean(iter_stats$y10)
```

```
        bias_y10 <- mean_y10 - mean(y1m0_all[,sce])
        rbias_y10 <- bias_y10 / mean(y1m0_all[,sce])
        bias2_y10 <- mean_y10 - y_pop[3, sce]
        mse_y10 <- mean((iter_stats$y10-y1m0_all[,sce])^2)
        sd_y10 <- sd(iter_stats$y10)
        meanse_y10 <- mean(iter_stats$se_y10)
        # y01 summary
        mean_y01 <- mean(iter_stats$y01)
        bias_y01 <- mean_y01 - mean(y0m1_all[,sce])
        rbias_y01 <- bias_y01/mean(y0m1_all[,sce])
        bias2_y01 <- mean_y01 - y_pop[4, sce]
        mse_y01 <- mean((iter_stats$y01-y0m1_all[,sce])^2)
        sd_y01 <- sd(iter_stats$y01)
        meanse_y01 <- mean(iter_stats$se_y01)

        ## Save all summary statistics of this data frame to one row
        sum_stats <-  c(sce, smp, wstrat, mean_swde,
                        mean_te, bias_te, rbias_te, bias2_te,mse_te, sd_te, meanse_te,
                        mean_nde0, bias_nde0, rbias_nde0, bias2_nde0, mse_nde0, sd_nde0, meanse_nde0,
                        mean_nie1, bias_nie1, rbias_nie1, bias2_nie1, mse_nie1, sd_nie1, meanse_nie1,
                        mean_nde1, bias_nde1, rbias_nde1, bias2_nde1, mse_nde1, sd_nde1, meanse_nde1,
                        mean_nie0, bias_nie0, rbias_nie0, bias2_nie0, mse_nie0, sd_nie0, meanse_nie0,
                        min_ess00, mean_ess00, max_ess00, min_ess11, mean_ess11, max_ess11,
                        min_ess10, mean_ess10, max_ess10, min_ess01, mean_ess01, max_ess01,
                        min_ess00_b,mean_ess00_b, max_ess00_b, min_ess11_b,mean_ess11_b, max_ess11_b,
                        min_ess10_b,mean_ess10_b, max_ess10_b, min_ess01_b,mean_ess01_b, max_ess01_b,
                        meanchi_treatmed, meanchi_treatout, meanchi_medout,
                        mean_y00, bias_y00, rbias_y00, bias2_y00, mse_y00, sd_y00, meanse_y00,
                        mean_y11, bias_y11, rbias_y11, bias2_y11, mse_y11, sd_y11, meanse_y11,
                        mean_y10, bias_y10, rbias_y10, bias2_y10, mse_y10, sd_y10, meanse_y10,
                        mean_y01, bias_y01, rbias_y01, bias2_y01, mse_y01, sd_y01, meanse_y01)
        return(sum_stats)
}
stopImplicitCluster()
end_time <- Sys.time()
end_time - start_time
```

```
# combine all the elements of sum_stats_list in sum_table
for (r in 1:128){
        sum_table[r,] <- sum_stats_list[[r]]
}

# export sum_table
write_csv(sum_table, file = "./sum_table.csv")
write_xlsx(sum_table, "./sum_table.xlsx")
end_time2 <- Sys.time()
end_time2 - start_time2

# check relative comparison of mean se and sd
# scenario 1, TE for example
range(sum_table[sum_table$scenario == 1, ]$meanse_te / sum_table[sum_table$scenario ==1,]$sd_te)



# check if there is bias in TE estimates under "both stages", Scenario 8
bias_te_844<- iter_stats_table[iter_stats_table$scenario == 8 &
                         iter_stats_table$weight_strategy == 4 &
                         iter_stats_table$sampling == 4, ]$bias_te
t.test(bias_te_844, mu = 0)




# Plots
library(ggplot2)
library(reshape2)
library(dplyr)
estimates_table <- iter_stats_table[, c("scenario", "weight_strategy", "sampling",
"bias_te", "bias_nie1", "bias_nde0", "bias_nie0", "bias_nde1")]
colnames(estimates_table)<- c("scenario", "weight_strategy", "sampling", "TE", "NIE1",
"NDE0", "NIE0", "NDE1")
sce_labs <- c("Scenario 1", "Scenario 8")
```

```
names(sce_labs) <- c("1", "8")
estimates_table_l <- melt(estimates_table, id.var = c("scenario", "weight_strategy", "sampling"))
# readjust factor levels of scenario
estimates_table_l$variable<-
        factor(estimates_table_l$variable,
               labels=c("NDE0","NIE0", "NDE1", "TE", "NIE1"))



# SD vs MeanSE
sd_table<- sum_table[, c("scenario", "sampling", "weight_strategy", "sd_te",
                            "sd_nie1", "sd_nde0", "sd_nie0", "sd_nde1")]
se_table <- sum_table[, c("scenario", "sampling", "weight_strategy", "meanse_te", "meanse_nie1",
                          "meanse_nde0", "meanse_nie0", "meanse_nde1")]
# long tables (l: long)
sd_table_l <- melt(sd_table, id.var = c("scenario", "sampling","weight_strategy"))
se_table_l <- melt(se_table, id.var = c("scenario", "sampling","weight_strategy"))
colnames(sd_table_l) <- c("scenario", "sampling","weight_strategy", "variable", "sd_value")
colnames(se_table_l) <- c("scenario", "sampling","weight_strategy", "variable", "meanse_value")
sd_table_l$variable <- sd_table_l$variable

sd_table_l$variable <- toupper(substr(sd_table_l$variable, start = 4, stop = 7))
se_table_l$variable <- toupper(substr(se_table_l$variable, start = 8, stop = 11))

# order the levels of te, nie, nde
estimates_table_l$variable <- factor(estimates_table_l$variable,
                                levels = c("NIE0","NDE1", "TE", "NIE1", "NDE0"))
## put all scenarios in a loop
estimates <- vector("list", length = 8)
plots_sce <- vector("list", length = 8)
names(plots_sce) <- c("plot_sce1", "plot_sce2", "plot_sce3", "plot_sce4",
                       "plot_sce5", "plot_sce6", "plot_sce7", "plot_sce8")
sce_labs <- c("Scenario 1", "Scenario 2","Scenario 3","Scenario 4",
              "Scenario 5","Scenario 6","Scenario 7","Scenario 8")
names(sce_labs) <- 1:8
for (sce in 1:8){
        estimates[[sce]]<- estimates_table_l[estimates_table_l$scenario ==sce,]
```

```
}
for (sce in 1:8){
        plots_sce[[sce]] <-
                ggplot(estimates[[sce]],
                       aes(x=sampling,y=value, fill=weight_strategy)) +
                geom_boxplot(outlier.size=.5)+
                #facet_grid(~variable)+
                facet_wrap(~variable, nrow = 2, as.table = FALSE)+
                labs(x = "Sampling Methods", y = "Estimates")+
                #theme_classic()+
                theme_bw()+
                theme(plot.title = element_text(size = 30),
                      axis.title=element_text(size=25),
                      axis.text = element_text(size=22),
                      legend.title = element_text(size = 25),
                      legend.text = element_text(size = 22),
                      strip.text = element_text(size = 20))+
                      # legend.position = "top")+
                geom_hline(yintercept=0, linetype="solid", color = "black")+
                # ggtitle(paste0("Distribution of Effect Estimates: ", sce_labs[sce]) ) +
                scale_fill_discrete(name = "Sampling weighting\n strategies",
                        labels = c("Neither stage", "2nd stage",
                                   "1st stage", "Both stages"))
}
# check results
plots_sce[1]
plots_sce[2]
plots_sce[3]
plots_sce[4]
plots_sce[5]
plots_sce[6]
plots_sce[7]
plots_sce[8]
```

```
#sd_table_l$id <- 1:nrow(sd_table_l)
#se_table_l$id <- 1:nrow(se_table_l)
sdse_table_L <- merge(sd_table_l, se_table_l, by = c("scenario", "sampling",
                                                     "weight_strategy", "variable"))

# adjust the order of effects in the plots
sdse_te_l <- sdse_table_L[sdse_table_L$variable == "TE",]
sdse_nie1_l <- sdse_table_L[sdse_table_L$variable == "NIE1",]
sdse_nde0_l <- sdse_table_L[sdse_table_L$variable == "NDE0",]
sdse_nie0_l <- sdse_table_L[sdse_table_L$variable == "NIE0",]
sdse_nde1_l <- sdse_table_L[sdse_table_L$variable == "NDE1",]
sdse_table_L <- rbind(sdse_te_l, sdse_nie1_l) %>%
  rbind(sdse_nde0_l) %>%
  rbind(sdse_nie0_l) %>%
  rbind(sdse_nde1_l)

# the SD vs mean(SE) plots for the appendix
sdse_table_L$variable <- factor(sdse_table_L$variable,
                            levels = c("TE", "NIE1", "NDE0", "NIE0", "NDE1"))
sdse_table_L$scenario <- factor(sdse_table_L$scenario)
sdse_sce1<- sdse_table_L[sdse_table_L$scenario == 1,]

sdse_tables <- vector("list", length = 8)
plots_sdse <- vector("list", length = 8)
# eight tables, each for a scenario's sdse_table
for (sce in 1:8){
        sdse_tables[[sce]] <- sdse_table_L[sdse_table_L$scenario == sce,]
        sdse_tables[[sce]]$variable <- factor(sdse_tables[[sce]]$variable,
                                               levels=c("NIE0","NDE1", "TE", "NIE1", "NDE0"))
}

# potential meanse_sd plots for the appendix
# all scenarios
plots_sdse <- vector("list", 8)
names(plots_sdse) <- c("plot_sdse1", "plot_sdse2", "plot_sdse3", "plot_sdse4",
                      "plot_sdse5", "plot_sdse6", "plot_sdse7", "plot_sdse8")
```

```
design <- "
        123
        #45
"
for (sce in 1:8){
        plots_sdse[[sce]] <- ggplot(sdse_tables[[sce]],
        aes(x=sd_value,y=meanse_value,col=weight_strategy,shape=sampling)) +
                facet_grid(scenario~variable, labeller = labeller(scenario = sce_labs))+
                facet_wrap(~variable, nrow = 2, as.table = FALSE)+
                # plot_layout(design = design)+
                #theme_classic()+
                theme_bw()+
                labs(x = "SD", y = "Mean SE")+
                theme(plot.title = element_text(size = 30),
                      axis.title=element_text(size=25),
                      axis.text = element_text(size=15),
                      legend.title = element_text(size = 25),
                      legend.text = element_text(size = 22),
                      strip.text = element_text(size = 20))+
                      # legend.position = "top")+
                geom_abline(intercept=0,slope=1,col="black")+
                # ggtitle(paste0("Estimate SD vs. Mean SE: ", sce_labs[sce],
                "\nPlotting symbols by Sampling Methods 1-4") ) +
                scale_color_discrete(name = "Sampling weighting\n strategies",
                                     breaks = c("1", "2", "3", "4"),
                                     labels = c("Neither stage", "2nd stage",
                                                "1st stage", "Both stages")) +
                scale_shape_manual(name = "Sampling Methods",
                                   values = c("1","2","3","4"),
                                   breaks = c("1", "2", "3", "4"),
                                   labels = c("1", "2",
                                              "3", "4"),
                                   guide="none")+
                geom_point(size = 5)
}
```

```
plots_sdse[1]
plots_sdse[2]
plots_sdse[3]
plots_sdse[4]
plots_sdse[5]
plots_sdse[6]
plots_sdse[7]
plots_sdse[8]



# a function to move legends to "wasted" white space
shift_legend <- function(p){

        # check if p is a valid object
        if(!"gtable" %in% class(p)){
                if("ggplot" %in% class(p)){
                        gp <- ggplotGrob(p) # convert to grob
                } else {
                        message("This is neither a ggplot object nor a grob generated
                        from ggplotGrob. Returning original plot.")
                        return(p)
                }
        } else {
                gp <- p
        }

        # check for unfilled facet panels
        facet.panels <- grep("^panel", gp[["layout"]][["name"]])
        empty.facet.panels <- sapply(facet.panels,
                                function(i) "zeroGrob" %in% class(gp[["grobs"]][[i]]))
        empty.facet.panels <- facet.panels[empty.facet.panels]
        if(length(empty.facet.panels) == 0){
                message("There are no unfilled facet panels to shift legend into.
                Returning original plot.")
                return(p)
```

```
        }

        # establish extent of unfilled facet panels (including any axis cells in between)
        empty.facet.panels <- gp[["layout"]][empty.facet.panels, ]
        empty.facet.panels <- list(min(empty.facet.panels[["t"]]), min(empty.facet.panels[["l"]]),
                                   max(empty.facet.panels[["b"]]), max(empty.facet.panels[["r"]]))
        names(empty.facet.panels) <- c("t", "l", "b", "r")

        # extract legend & copy over to location of unfilled facet panels
        guide.grob <- which(gp[["layout"]][["name"]] == "guide-box")
        if(length(guide.grob) == 0){
                message("There is no legend present. Returning original plot.")
                return(p)
        }
        gp <- gtable_add_grob(x = gp,
                              grobs = gp[["grobs"]][[guide.grob]],
                              t = empty.facet.panels[["t"]],
                              l = empty.facet.panels[["l"]],
                              b = empty.facet.panels[["b"]],
                              r = empty.facet.panels[["r"]],
                              name = "new-guide-box")

        # squash the original guide box's row / column (whichever applicable)
        # & empty its cell
        guide.grob <- gp[["layout"]][guide.grob, ]
        if(guide.grob[["l"]] == guide.grob[["r"]]){
                gp <- gtable_squash_cols(gp, cols = guide.grob[["l"]])
        }
        if(guide.grob[["t"]] == guide.grob[["b"]]){
                gp <- gtable_squash_rows(gp, rows = guide.grob[["t"]])
        }
        gp <- gtable_remove_grobs(gp, "guide-box")

        return(gp)
}
```

```
plots_sce <- lapply(lapply(plots_sce, shift_legend), as.ggplot)
plots_sdse <- lapply(lapply(plots_sdse, shift_legend), as.ggplot)

# export the distribution box plots
for (i in 1:length(plots_sce)){
        ggsave(paste0("./plot_appendix/", names(plots_sce)[i], ".png"),
               plots_sce[[i]], width = 15, height = 10)
}

for (i in 1:length(plots_sdse)){
        ggsave(paste0("./plot_appendix/", names(plots_sdse)[i], ".png"),
               plots_sdse[[i]], width = 15, height = 10)
}
```
